# Large-Scale Chemical Similarity Networks for Target Profiling of Compounds Identified in Cell-Based Chemical Screens

**DOI:** 10.1371/journal.pcbi.1004153

**Published:** 2015-03-31

**Authors:** Yu-Chen Lo, Silvia Senese, Chien-Ming Li, Qiyang Hu, Yong Huang, Robert Damoiseaux, Jorge Z. Torres

**Affiliations:** 1 Department of Chemistry and Biochemistry, University of California, Los Angeles, Los Angeles, California, United States of America; 2 Program in Bioengineering, University of California, Los Angeles, Los Angeles, California, United States of America; 3 Drug Studies Unit, Department of Bioengineering & Therapeutic Sciences, University of California, San Francisco, San Francisco, California, United States of America; 4 Institute for Digital Research and Education, University of California, Los Angeles, Los Angeles, California, United States of America; 5 California NanoSystems Institute, University of California, Los Angeles, Los Angeles, California, United States of America; 6 Jonsson Comprehensive Cancer Center, University of California, Los Angeles, Los Angeles, California, United States of America; 7 Molecular Biology Institute, University of California, Los Angeles, Los Angeles, California, United States of America; Hellas, GREECE

## Abstract

Target identification is one of the most critical steps following cell-based phenotypic chemical screens aimed at identifying compounds with potential uses in cell biology and for developing novel disease therapies. Current *in silico* target identification methods, including chemical similarity database searches, are limited to single or sequential ligand analysis that have limited capabilities for accurate deconvolution of a large number of compounds with diverse chemical structures. Here, we present CSNAP (Chemical Similarity Network Analysis Pulldown), a new computational target identification method that utilizes chemical similarity networks for large-scale chemotype (consensus chemical pattern) recognition and drug target profiling. Our benchmark study showed that CSNAP can achieve an overall higher accuracy (>80%) of target prediction with respect to representative chemotypes in large (>200) compound sets, in comparison to the SEA approach (60–70%). Additionally, CSNAP is capable of integrating with biological knowledge-based databases (Uniprot, GO) and high-throughput biology platforms (proteomic, genetic, etc) for system-wise drug target validation. To demonstrate the utility of the CSNAP approach, we combined CSNAP's target prediction with experimental ligand evaluation to identify the major mitotic targets of hit compounds from a cell-based chemical screen and we highlight novel compounds targeting microtubules, an important cancer therapeutic target. The CSNAP method is freely available and can be accessed from the CSNAP web server (http://services.mbi.ucla.edu/CSNAP/).

## Author Summary

Determining the targets of compounds identified in cell-based high-throughput chemical screens is a critical step for downstream drug development and understanding of compound mechanism of action. However, current computational target prediction approaches like chemical similarity database searches are limited to single or sequential ligand analyses, which limits their ability to accurately deconvolve a large number of compounds that often have chemically diverse structures. Here, we have developed a new computational drug target prediction method, called CSNAP that is based on chemical similarity networks. By clustering diverse chemical structures into distinct sub-networks corresponding to chemotypes, we show that CSNAP improves target prediction accuracy and consistency over a board range of drug classes. We further coupled CSNAP to a mitotic database and successfully determined the major mitotic drug targets of a diverse compound set identified in a cell-based chemical screen. We demonstrate that CSNAP can easily integrate with diverse knowledge-based databases for on/off target prediction and post-target validation, thus broadening its applicability for identifying the targets of bioactive compounds from a wide range of chemical screens.

This is a *PLOS Computational Biology* Methods article.

## Introduction

The use of chemical screens to identify molecules for the treatment of proliferative diseases like cancer has relied on two major strategies, target-based screening and phenotypic screening [1,2]. Unbiased cell-based screens, including phenotypic screens, have successfully discovered numerous cytotoxic agents that inhibit cancer cell proliferation. By assaying structurally diverse compounds, cell-based phenotypic chemical screens have the potential to discover a multitude of druggable protein targets that modulate cell cycle progression through diverse mechanisms [2]. However, a major hurdle for cell-based phenotypic chemical screens has been the deconvolution of active compounds, i.e. target identification [2,3]. Classical methods for target identification like chemical proteomics rely on compound modification and immobilization to generate compound affinity matrixes that can be used to pull down associated proteins [4]. Without prior knowledge of compound structure-activity-relationship (SAR), the modification of key functional groups can occlude compound activity and hamper protein-ligand interactions [5]. Additionally, these approaches are labor intensive, costly and have a low success rate.

Computational approaches for predicting the targets, off-targets and poly-pharmacology of hit compounds have been used widely in recent years due to their speed, flexibility and ability to be easily coupled to experimental validation techniques [1,2]. *In-silico* target inference methods include ligand-based and structure-based approaches. Ligand-based approaches, such as similarity ensemble approach (SEA), SuperPred, TargetHunter, HitPick, ChemMapper and others, compare hit compounds to a database of annotated compounds and drug targets of hit compounds are inferred from the targets of the most similar annotated compounds, based on their chemical structure similarity [6–9]. The premise of the 2D chemical similarity inference approach is the “chemical similarity principle”, which states that structurally similar compounds likely share similar biological activities [10–12]. The efficiency of 2D chemical search algorithms also led to the wide adoption of this target inference method in public bioactivity database searches including ChEMBL and PubChem [13,14]. Recently, similarity-based target inference has been extended to incorporate 3D chemical descriptors derived from the bioactive conformations of molecules [15]. For example, PharmMapper, ROCS and the Phase Shape programs use a reverse pharmacophore and shape matching strategy to identify putative targets [16–18]. Albeit computationally intensive, a major advantage of this approach is that “scaffold-hoppers” can be deorphanized, as these compounds often share low chemical similarity but bind similarly to known receptor sites [19]. On the other hand, structure-based target inference approaches, such a TarFisDock and INVDOCK, apply reverse panel docking and ranking of docking scores to predict protein targets from pre-annotated structures [10,20]. In comparison, ligand-based approaches are particularly advantageous due to their speed and algorithmic simplicity and they are not limited by structure availability. However, current ligand-based approaches analyze bioactive molecules in an independent sequential fashion, which has several disadvantages [2,8,21]. For example, target inference is based on finding a single most similar annotated compound for a given query ligand, which may not provide consistent target prediction for a group of structurally similar ligands. Additionally, subtle structural changes in the functional groups of active molecules can alter their potency and specificity toward drug targets; thus, analyzing each molecule independently may not offer a coherent SAR for a congeneric series. This suggests that a more global and systematic analysis of compound bioactivity is required to improve the current state of *in-silico* drug target prediction.

Several global approaches to drug target profiling have been developed [2]. One approach is bioactivity profile matching, where model organisms are treated with compounds and compounds that induce similar phenotypic responses are clustered and inferred to have similar mechanisms of action [2,22,23]. However, bio-signature fingerprint comparisons do not infer direct protein-ligand interactions. Furthermore, large numbers of measurements are required to construct such fingerprints [22,24]. Alternatively, computational networks have been effectively utilized to mine the existing protein-ligand interaction data deposited in bioactivity databanks. One example is the drug-target network (DTN), which utilizes a bipartite network encompassing interconnecting ligand and target vertex to capture complex poly-pharmacological interactions [25]. While this prediction model is useful for predicting drug side effects and identifying novel protein-ligand pairs, DTN demands statistical learning from prior protein-ligand interaction data using Beyesian analyses or Support Vector Machines. Thus, DTN’s predictability beyond the training space may not be accurate, limiting DTN’s applicability for large-scale drug target prediction [26–29].

To address the current challenges in computational drug target prediction, we developed a new drug target inference approach based on chemical similarity networks (CSNs) and implemented this approach as a computational program called CSNAP (Chemical Similarity Network Analysis Pull-down). CSN is a promising computational framework that allows large-scale SAR analysis by clustering compounds based on their structural similarity [30]. This framework has recently been applied to investigate “bioactivity landscapes” from known drugs as well as for analyzing bioactivity correlations among secondary metabolites [30,31]. Furthermore, several network characteristics including degree of connectivity, centrality and cohesiveness offer critical information to study the global topology of large chemical networks and allow key compound members to be identified [32,33]. Although CSNs have been widely applied to SAR studies, their application to drug target inference has not been explored [30,32]. In our CSNAP approach, both query and annotated compounds are first clustered into CSNs, where nodes represent compounds and edges represent chemical similarity. The target annotations of the reference nodes are assigned to the connecting query nodes whenever two node types form a chemical similarity edge above a similarity threshold [13,34,35]. To determine the most probable target, a consensus statistics score is determined by the target annotation frequency shared among the immediate neighbors (first-order neighbor) of each query compound in the network. When multiple ligands were analyzed by the CSNAP approach, diverse compound structures were clustered into distinct chemical similarity sub-networks corresponding to a specific “chemotype” (i.e. consensus chemical scaffold), which was associated with specific drug targets [36]. Within the context of drug design, “chemotype” has been widely used for drug repurposing. For example, a single scaffold can be diversified by combinatorial synthesis to modulate its specificity toward multiple secondary targets [36]. On the other hand, the CSNAP approach identifies consensus “chemotypes” from diverse chemical structures, which likely inhibit common targets capable of inducing similar phenotypes in cell culture. In contrast to current target prediction methods, CSNAP does not rely on absolute chemical similarity nor does it necessitate a training set to make target inferences. Additionally, CSNAP is capable of integrating with chemical and biological knowledge-based databases (Uniprot, GO) and high-throughput biology platforms (proteomic, genetic, etc) for system-wise drug target validation. Our benchmark study showed that CSNAP can achieve an overall higher accuracy (>80%) of target prediction with respect to representative chemotypes in large (>200) compound sets, in comparison to the SEA approach (60–70%). To demonstrate the utility of the CSNAP approach, we combined CSNAP's target prediction with experimental ligand evaluation to identify the major mitotic targets of hit compounds from a cell-based chemical screen and we highlight novel compounds targeting microtubules, an important cancer therapeutic target. The CSNAP method is freely available and can be accessed from the CSNAP web server (http://services.mbi.ucla.edu/CSNAP/).

## Results

### CSNAP workflow

**Fig 1:**
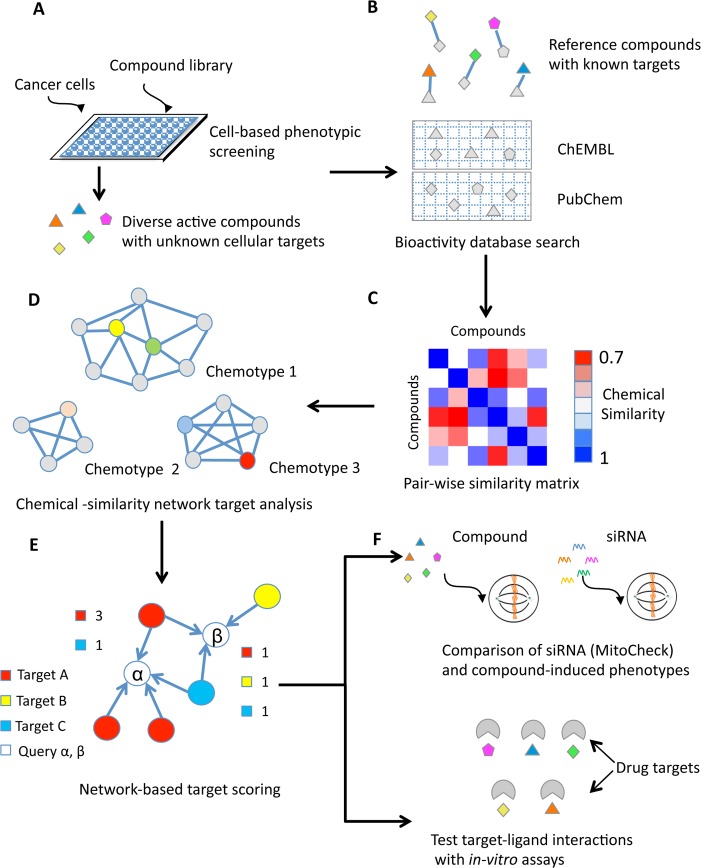
Overview of the CSNAP approach for high-throughput compound target identification using Chemical Similarity Networks (CSNs). (A) Discovery of diverse ligands from cell-based screens with unknown cellular targets. Note that structurally distinct compound classes are represented by different shapes, while structurally-related analogs within each class are labeled with different colors. (B) Target identification using CSNAP. Bioactivity database searches to identify structurally similar reference compounds with known target annotations. The grey nodes represent target annotated compounds. (C) A pair-wise similarity matrix was computed by considering both intra and inter-ligand similarity between query and reference compounds using Tanimoto coefficient (Tc) with cutoff > 0.7. (D) Structurally diverse ligands are clustered into chemical similarity subnetworks based on representative chemotypes (consensus chemical patterns). (E) The network topology was used to guide and quantify the protein-ligand interactions for drug target prediction. Two neighbor counting functions, S-score and H-score were applied to identify and rank the most common targets among the first-order neighbors of the query compounds within the CSN. In this example, compound α has a consensus Target A score = 3 and a Target C score = 1, whereas compound β has a consensus score = 1 for Target A, B and C. (F) Experimental target validation. The predicted targets were validated by comparing RNAi with compound-induced cellular phenotypes and by testing direct protein-ligand interactions in *in-vitro* assays.

We have developed a new computational workflow for compound target deconvolution and prioritization of compounds based on chemical similarity networks that we have termed CSNAP (Chemical Similarity Network Analysis Pull-down) (Fig. 1). In CSNAP, the Obabel FP2 fingerprints, which characterize molecules by a series of structural motifs as binary numbers (0 and 1), were utilized for structural comparison and compound retrieval from the ChEMBL database (version 16) containing more than 1 million annotated molecules with reported bioactivities (Fig. 1A, 1B and S1 Text) [13,37]. In comparison to other available fingerprints (FP3, FP4 and MACCS), the FP2 fingerprint uses a path-based algorithm, which has high specificity, is generally applicable to any ligand size and is not limited to pre-defined substructure patterns [38]. To retrieve structurally similar ligands from the bioactivity database, two chemical similarity search functions were used: a threshold similarity search based on a Tanimoto coefficient (Tc) score and a Z-score (S1 Text) [39,40]. The Tc score is one of the most commonly used metrics for chemical similarity comparison in chemoinformatics, which compares two chemical fingerprints to determine the fraction of shared bits with values ranging from 0 to 1. However, a fixed similarity threshold search may not detect compounds with statistical significant scores; thus, a Z-score was also used to search database compounds based on the overall similarity score distribution of the hits [40]. The target annotations of the selected ChEMBL compounds (baits) most similar to input ligands were subsequently retrieved from the ChEMBL and PubChem databases (Fig. 1B and S1 Text). Based on the output of ligand similarity comparisons, a chemical similarity network was constructed by connecting pairs of ligands with similarity above a Tc threshold according to a weighted adjacency matrix (Fig. 1C and S1 Text) [41]. This resulted in weighted graphs (networks) in which nodes represent compounds and edges represent chemical similarity (Fig. 1D).

Target inference of the query compounds within the CSNAP-generated network, which contains both query and reference nodes, is similar to the protein functional assignment in protein-protein interaction (PPI) networks, where protein functional lineage between a characterized and an uncharacterized protein are used to assign shared protein functions [34,42]. Multiple scoring schemes have been developed to infer protein functions in PPI networks, including algorithms based on network connectivity, graph topology and modular recognition [43–45]. The most direct network-based scoring scheme is the neighbor counting method, where the annotation frequency in the immediate neighbors is ranked and assigned to the linked queries. Thus, the similarity between PPI networks and CSNs suggested that this approach could be effective for network-based drug target inference. As a proof-of-principle, we applied two neighbor-counting functions, Schwikowski score and Hishigaki score for drug target prediction in CSNAP networks [43,46]. Specifically, a target consensus statistics score, Schwikowski score (S-score), was calculated by ranking the most common targets shared among the neighboring annotated ligands of each query compound within the network (Fig. 1E and S1 Text) [43]. Additionally, a Hishigaki score (H-score), a chi-square like test based on the mean target annotation frequency distributed within the whole network, was also implemented to compute a significance value for each drug target assignment (S1 Text) [46]. The rationale for applying Schwikowski and Hishigaki scoring functions in CSNAP target inference, apart from their algorithmic efficiency and scalability for large-scale network computation, was their accuracy. For example, it was shown that a Schwikowski score correctly predicted >70% of proteins with at least one functional category in a large-scale *S*. *cerevisiae* PPI network [43]. Furthermore, a performance comparison in a *S*. *cerevisiae* network showed that these nearest neighbor approaches offer high specificity and prediction accuracy, making them competitive against more advanced statistical network models including Markov random field (MRF) and kernel logistic regression [33,34].

### CSNAP validation using benchmark compounds

**Fig 2:**
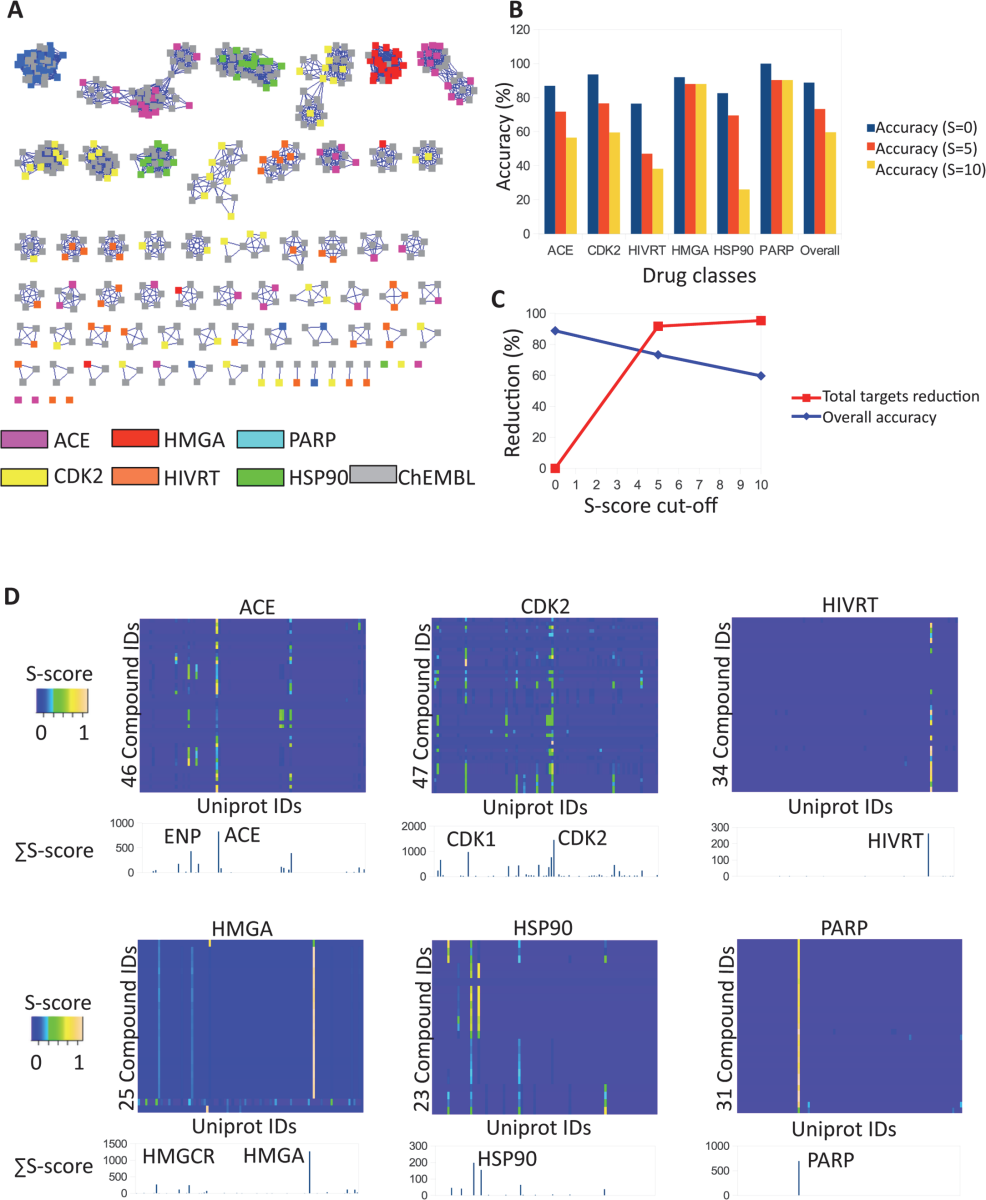
CSNAP validation using benchmark compounds. (A) 206 compounds from six major drug classes (ACE, CDK2, HIVRT, HMGA, HSP90 and PARP) were analyzed using CSNAP with a Z-score cutoff of 2.5 and a Tanimoto coefficient (Tc) cutoff of 1. With the exception of 7 molecules, all compounds were ordered into chemical similarity subnetworks specific to each drug target. (B) Outcome of applying the neighbor counting function, S-score to predict the top 5 most common targets shared by the annotated-neighbor nodes of all input ligands within the CSN. The prediction accuracy (percentage of correctly predicted ligands) was determined by comparing the predicted target to ligand target annotations. CSNAP target prediction assessment for each drug class ranked by different S-score cutoffs (S-cutoff = 0, 5 and 10) gave an overall prediction accuracy of 89%, 73% and 60% respectively. (C) Comparison of the total percentage of target pool reduction (percentage of the total number of predicted targets with S-score cutoff over total number of predicted targets with S-score cutoff) against the overall prediction accuracy indicated that an S-score cutoff of 4 is optimal for hit enrichment and target virtual screening. (D) CSNAP target and off-target prediction for benchmark compounds. Predicted targets for the validation compounds were plotted against each drug class to identify targets and off-targets using Ligand-Target Interaction Fingerprints (LTIFs) analyzed on heat maps. The color intensity was scaled according to the S-score (0–1). Note that ACE and CDK2 inhibitors have predicted off-targets based on the additional coloring patterns, indicating drug poly-pharmacology. See S1 Fig for LTIF analysis of the combined drug classes.

To validate CSNAP computationally, we tested CSNAP’s ability to correctly predict the assigned targets for annotated compounds as well as its ability to cluster compounds with similar target specificities using a diversity set retrieved from the directory of useful decoys (DUD LIB VS 1.0) [47]. The diversity set contained 206 ligands from 6 target-specific drug classes with known target annotations (including 46 angiotensin-converting enzyme (ACE), 47 cyclin-dependent kinase 2 (CDK2), 23 heat-shock protein 90 (HSP90), 34 HIV reverse-transcriptase (HIVRT), 25 HMG-CoA reductase (HMGA) and 31 Poly [ADP-ribose] polymerase (PARP) inhibitors) (S1 Table). Two chemical search criteria were initially tested for CSNAP drug target prediction including one search with a Z-score cutoff = 2.5 and Tc cutoff = 1 (identical match) and another search with a Z-score cutoff = 2.5 and Tc cutoff = 0.85. In comparison, using an absolute Tc similarity cutoff = 0.85 substantially increased the network density (number of nodes in each network cluster) but did not significantly affect the number of network clusters generated (66 and 61) (Figs 2A, S1 and S1 Text). In both cases, CSNAP was able to resolve 206 compounds into target specific chemical similarity sub-networks. Based on the chemical similarity network generated by the latter chemical search criteria, we then assessed the prediction accuracy (percentage of correctly predicted ligands) for each drug class by considering the top five consensus targets ranked by S-scores; meanwhile, we applied a set of S-score cutoffs for hit enrichment to reduce the target pool (Fig. 2B, 2C and S1 Text). The results indicated that CSNAP’s overall prediction accuracy (recall-like score) for the benchmark compounds was 89% (S-score = 0) and 80% (S-score > = 4) respectively (Fig. 2B and 2C). Of those compounds with a prediction, the precision-like score was 94% (S-score = 0) and 85% (S-score > = 4) respectively.

To identify potential off-targets for these characterized drugs, we mapped the compound S-score for each drug class against the predicted targets using a ligand-target interaction fingerprint (LTIF), which allowed us to differentiate primary targets from off-targets on a heatmap (Fig. 2D and S1 Text) [48]. To further rank the most common targets within the whole compound set, we generated a target spectrum by summing the target prediction score, S-score for each predicted target, by which the heights of the target spectrum can be correlated with the total S-score (∑ S-score). Next, we identified the most probable targets and off-targets from the top peaks above the average ∑ S-score. While we cannot exclude smaller peaks as false positives, as they may represent an experimentally verified interaction of the reference compounds in the ChEMBL database, the higher peaks nevertheless represent the most common targets and off-targets among the analyzed ligands. Within the context of a chemical screen, additional target selection can be aided by gene ontology (GO) analysis, where molecular functions, cellular processes and pathway information can be used to verify the functional role of the predicted targets (see CSNAP website for additional details).

We subjected the diversity set to two different LTIF analyses, first by analyzing each drug class independently and then all drug classes combined. Independent LTIF analysis of HIVRT, HMGA and PARP compound sets revealed specific target binding patterns in contrast to CDK2 and ACE, which showed multiple interactions, suggesting potential off-target bindings (Fig. 2D). From the target spectrum, we identified ENP and CDK1 as the major off-targets for ACE and CDK2 inhibitors respectively, which had been previously reported (Fig. 2D) [49,50]. For the combined analysis, the targets and off-targets of the 206 benchmark compounds were likewise successfully identified from the target spectrum (S2 Fig). Although these validated compounds were “drug-like” and had been optimized for target specificity and transport properties, CSNAP analysis nevertheless identified potential off-targets that were not originally intended for these ligands. This indicated that CSNAP could potentially be used for high-throughput target deorphanization and off-target prediction for bioactive compounds from any chemical screen.

**Fig 3:**
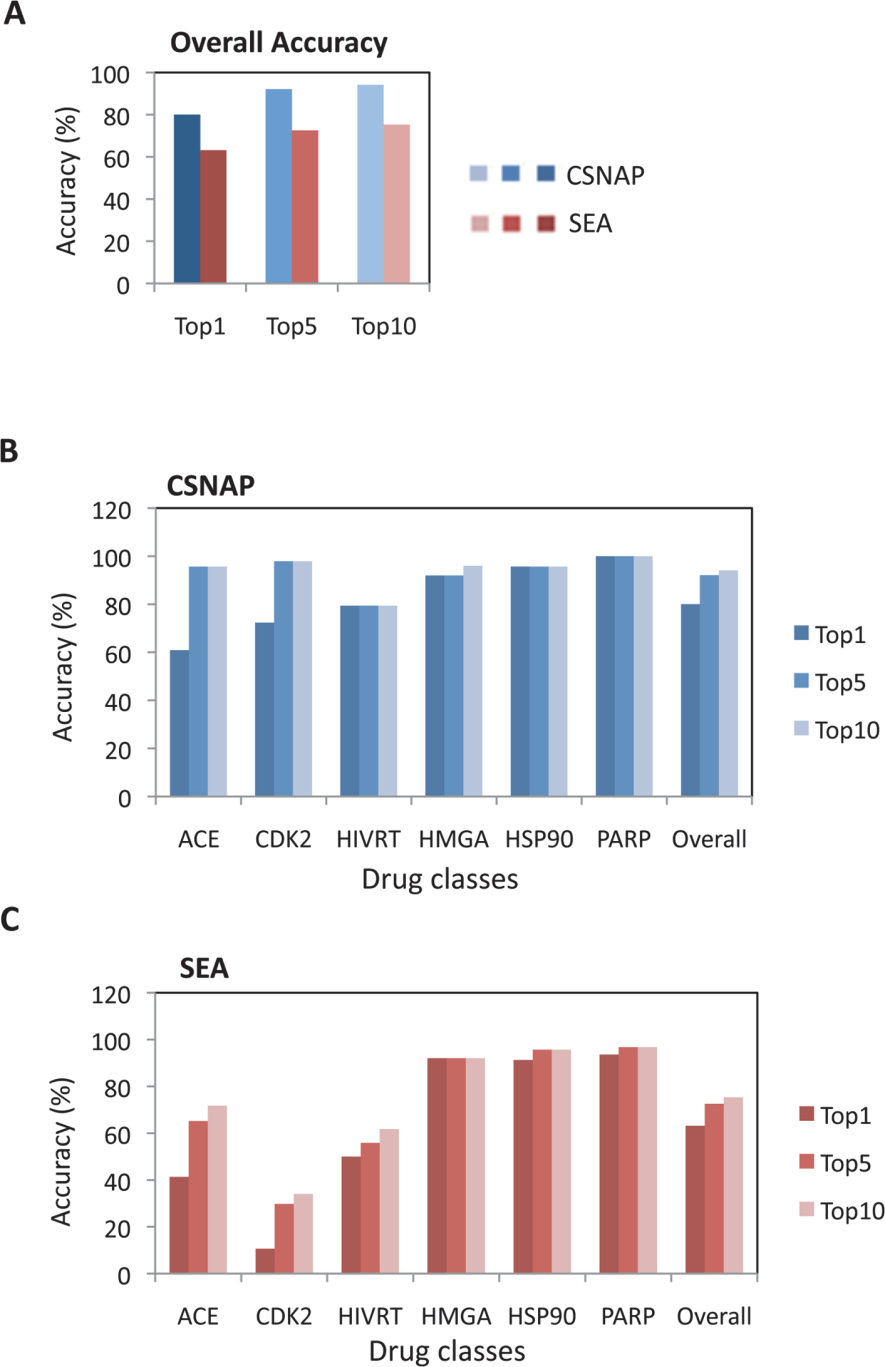
Target prediction accuracy comparison of network-based and ligand-based approaches. (A) Comparison of the overall target prediction accuracy based on the top hit, top five hits and top ten hits analyzed by CSNAP or the SEA approach using 206 benchmark compounds comprised of six major drug classes (ACE, CDK2, HIVRT, HMGA, HSP90 and PARP). The result shows that CSNAP provides a substantial improvement in target prediction accuracy over the traditional ligand-based approach by pair-wise chemical similarity comparison. (B and C) Detailed target prediction accuracy comparison breakdown of each of the six drug classes predicted by (B) CSNAP and (C) SEA approach respectively. The comparison showed that CSNAP provided a greater success rate at identifying the major targets of promiscuous ligands such as CDK2 and ACE inhibitors, which resulted in low prediction accuracies by the traditional ligand-based method.

Next, we compared CSNAP’s target prediction accuracy with SEA (Similarity Ensemble Approach), a widely used ligand-based target prediction approach based on sequential chemical similarity comparisons, to correctly identify the annotated targets of the benchmark sets (S1 Table and S1 Text) [51]. CSNAP showed an overall improvement in prediction accuracy (80–94%) over SEA (63–75%) at identifying the labeled targets of each of the six drug classes from the top 1, top 5 and top 10 score rankings by each respective method. In particular, CSNAP provided substantially better target prediction for promiscuous ligands such as CDK2 and ACE inhibitors (92% and 96%) than the SEA approach (30% and 65%) (Fig. 3A–3C and S1 Text).

### Target prediction of mitotic compounds from chemical screen

**Fig 4:**
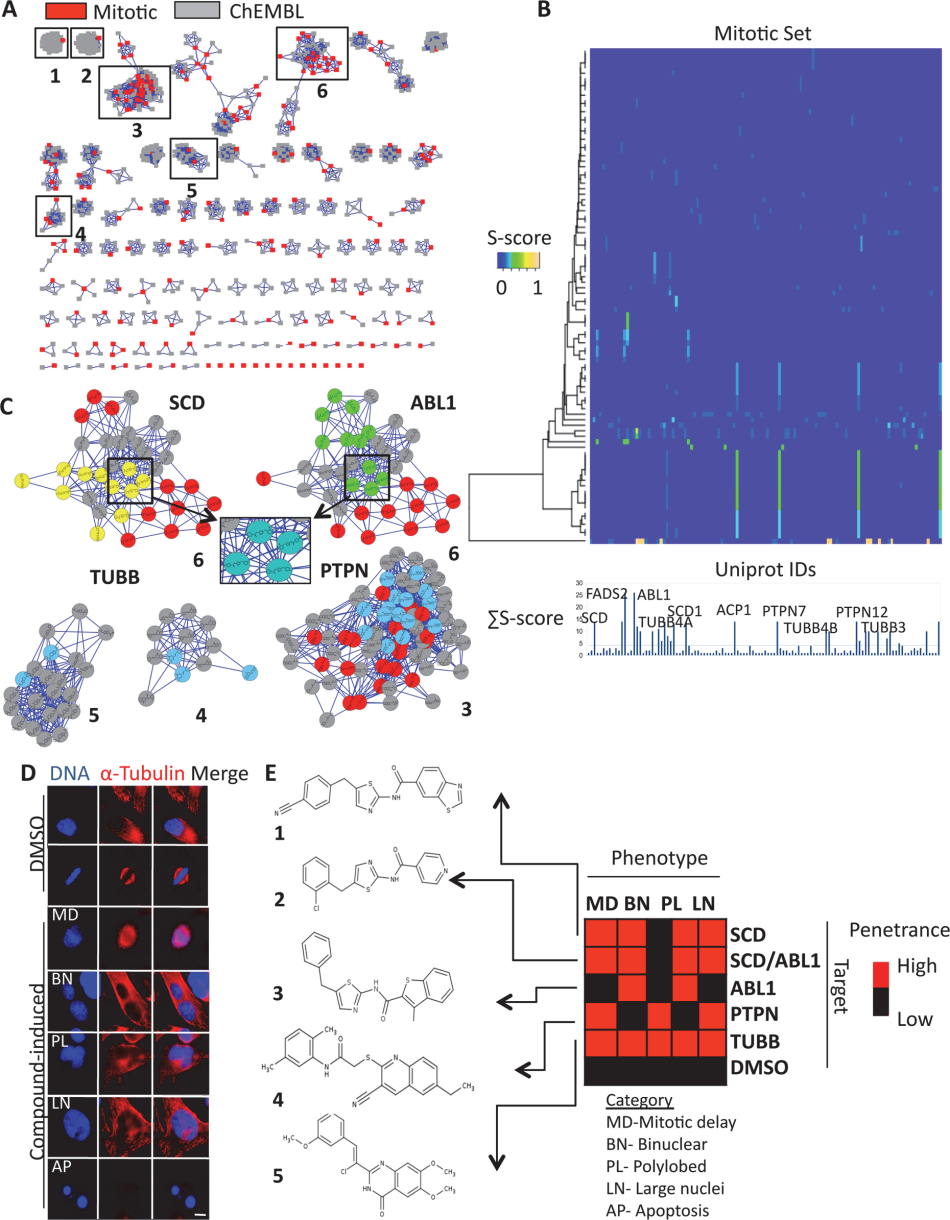
Integration of CSNAP with knowledge databases for mitotic target prediction and phenotypic target validation. (A) Mitotic compound chemical similarity network. CSNAP analysis of 212 mitotic compounds yielded 85 chemical similarity clusters representing diverse chemotypes, only 21 compounds were not clustered into annotated similarity graphs. (B) LTIF analysis of CSNAP mitotic target predictions. The target spectrum identified four major classes of targets from the top peaks including fatty acid desaturase (SCD), ABL kinase (ABL1), phosphatase (PTPN) and tubulin (TUBB). An independent LTIF analysis of each target class is presented in S2 Fig. (C) Mitotic compound deconvolution. Target associated chemical similarity sub-networks of four predicted targets (SCD, ABL1, PTPN and TUBB) were “pulled-down” from the mitotic CSN. For each cluster, at least one mitotic compound connected to one or more reference nodes with Tc threshold> 0.7. Note that the predicted SCD and ABL1 compounds display over-lapping neighbors, indicating that the predicted targets may be modulated by both compound sets. (D) Phenotypic validation of predicted mitotic targets. Asynchronous HeLa cells were treated with indicated compounds for 24 hours, fixed and stained for DNA and Tubulin. The observed compound-induced cell division defects were compared to target gene expression knockdown defects within the MitoCheck database. All compounds matched the previously characterized phenotypes associated with knockdown of target protein expression. See S6 Fig for complete compound-induced phenotypes.

Recently, we performed a high-throughput cell-cycle modulator screen with a diverse, unbiased set of 90,000 drug-like compounds, which identified compounds arresting cancer cells in mitosis (212 compounds) (S2, S3 Tables and S1 Text). We applied CSNAP to identify the potential targets of the 212 antimitotic compounds (S3 Fig and Supporting File). CSNAP analysis generated 85 chemical similarity sub-networks representing diverse chemotypes and retrieved 116 UniProt target IDs from ChEMBL annotations (Fig. 4A). These targets were analyzed using LTIF with a predefined cutoff (∑ S-score >10) from which we identified 4 broad categories of putative mitotic targets (20 UniProt target IDs) (Fig. 4B). These included 3 fatty acid desaturases (SCD, SCD1 and FADS2), 1 ABL1 kinase, 5 non-receptor type tyrosine phosphatases (PTPN7, PTPN12, PTPN22, PTPRC and ACP1) and 11 tubulin isoforms. Further compound deconvolution with respect to these targets identified 7 SCD inhibitors, 9 ABL1 inhibitors, 14 PTPN inhibitors and 7 TUBB inhibitors from 6 distinct clusters from the mitotic compound network (including SCD/ABL1: cluster 6, PTPN: cluster 3 and TUBB: clusters 1, 2, 4 and 5) and in which 4 compounds were shown to target both SCD and ABL1 (Figs 4C, S4 and S1 Text). Meanwhile, by querying the PubChem target annotations with respect to these four target categories, we identified an additional 19 tubulin-associated clusters (total 23), including 51 compounds with unknown bioactivities, which were predicted to be tubulin binders that covered ~20% of our mitotic set (S5A Fig). Among the predicted targets were the tubulins (TUBB, including α and β-tubulin), which are the building blocks of microtubules that are essential for mitotic spindle assembly and are established anticancer drug targets [52,53]. Consistently, several well-known microtubule-targeting agents were identified in the TUBB clusters including mebendazole and nocodazole from cluster 5 (Fig. 4A) [52]. Although the compound chemotypes for ABL1, SCD1 and PTPN were known, either identical or analogous to reference compounds deposited in the bioactivity databases, the assay context from which these compounds were retrieved was not related to mitosis [54–56]. Additionally, the function of ABL1, SCD1 and PTPN in mitotic progression had not been explored [57–60]. Thus, this analysis linked these proteins to potentially important new roles during cell division.

### Target validation of mitotic compounds from CSNAP predictions

To further substantiate that these compounds were likely inhibiting these targets (ABL1, SCD, PTPN and TUBB), we compared the phenotypes induced by their siRNA knockdown (which often correlates with inhibition of protein activity) with the phenotypes induced upon treatment with compounds from each target category using immunofluorescence (IF) microscopy [61]. To determine the target siRNA phenotype, we queried the MitoCheck database, which maintains data on the mitotic phenotypes observed upon siRNA knockdown of gene expression for most human genes (S1 Text). As expected, all four target categories (SCD, ABL1, PTPN and TUBB) displayed diverse mitotic defects by siRNA treatment [62]. This included defects in spindle assembly, chromosome segregation and cytokinesis that led to mitotic delay, post-mitotic defects (binuclear and polylobed nucleus) and apoptosis (cell death), suggesting that these targets were critical for cell division (S6 and S7 Figs) [62]. Next, five compounds from these target clusters were selected for phenotypic comparison including compound **1** from the SCD sub-cluster (cluster 6), compound **2** that overlapped with both SCD and ABL1 sub-clusters (cluster 6) and compound **3** from the ABL1 sub-cluster (cluster 6). Additionally, compound **4** and compound **5**, were retrieved from the PTPN cluster (cluster 3) and the TUBB cluster (cluster 4) respectively (Fig. 4A, 4C, and S4 Table). All five compounds showed consistent cell phenotypes between siRNA knockdown and drug treatment (Figs 4D, 4E, and S8). However, compound **1** (SCD sub-cluster) also displayed a “large nuclei” phenotype that was specific to ABL1 inhibitors, indicating that it may also target ABL1 based on chemical and phenotypic similarity (Fig. 4D, 4E, and S8). As expected, compound **2** (SCD/ABL1 sub-clusters) exhibited a “mixed” phenotype similar to compound **1** while compound **3** was ABL1 specific with very few mitotic delay and apoptotic cells that were specific to SCD inhibitors (Figs 4D, 4E, and S8).

**Fig 5:**
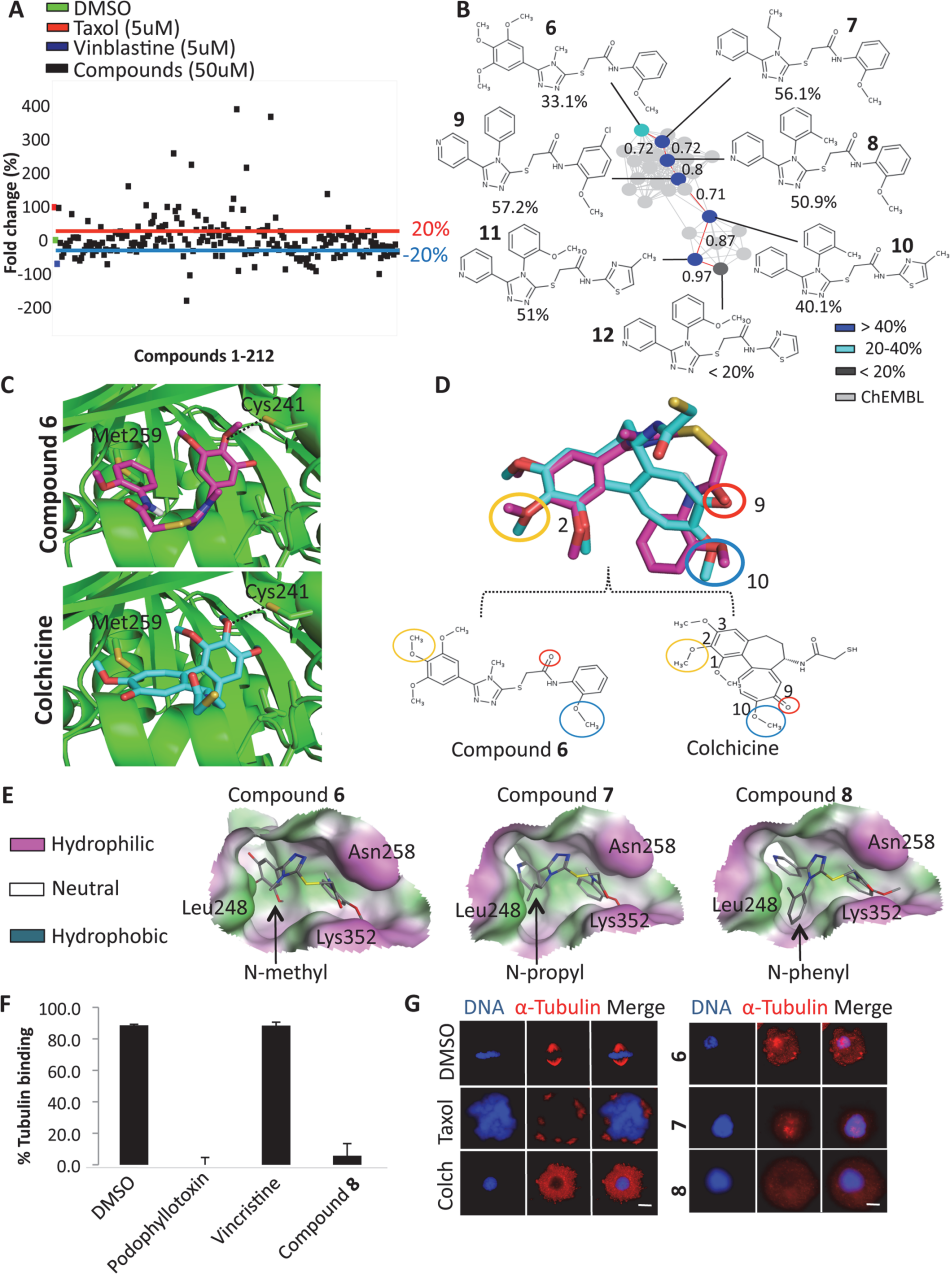
Network-based elucidation of a novel tubulin-targeting chemotype. (A) *In-vitro* tubulin polymerization assays were used to test the effect of the 212 mitotic compounds on microtubule assembly at 50μM concentration. The end-point absorbance, based on change in OD (dOD), was used to quantify the degree of microtubule polymerization and was converted to percentage fold change relative to DMSO (0%). Among the tested compounds, 134 compounds (63%) had an effect (>20% fold change) on tubulin polymerization. (B) Chemical similarity sub-network consisting of 7 novel anti-tubulin ligands based on a phenyl-sulfanyl-thiazol-acetamide privilege scaffold. The connected analogues within the network showed a consensus tubulin destabilization effect where each step in the path (red) of the sub-network corresponded to a minimum structural change correlating with the observed structure-activity-relationship (SAR). (C) Docking of compound **6** into the β-tubulin colchicine-binding site based on the crystal structure (PDB: 1AS0) exhibited a similar predicted binding mode to colchicine. (D) Ligand alignment between compound **6** and colchicine identified a conserved pharmacophore critical for ligand binding, including the 2 and 10-methoxy groups and a 9-keto group that interacts with Cys-241 of beta tubulin and Val-181 (not shown) of alpha tubulin respectively. (E) Hydrophobicity map of docked compound **6** within the colchicine-binding site revealed a hydrophobic sub-pocket enclosed by Leu-248 and Lys-352. The model showed that compounds **7** and **8** enhance binding affinity by fitting the N-propyl and N-phenyl group in the hydrophobic cavity, consistent with the SAR analysis. See S11 Fig for molecular modeling of compounds **6–12**. (F) The most potent compound **8** was tested for direct colchicine site binding using mass spectrometry competitive binding assays. Compound **8** competed strongly with colchicine for the colchicine-binding site, similar to the colchicine-site binder podophyllotoxin. Note that the negative control vincristine did not compete. (G) Immunofluorescence microscopy images of HeLa cells treated with DMSO, taxol, colchicine or compounds **6–8** for 20 hours. Cells were fixed and stained for DNA (Hoechst 33342) and tubulin (primary rat anti-tubulin antibodies and secondary anti-rat Cy3 antibodies). Scale = 5 μm. Note that colchicine, and compounds **6–8** depolymerize microtubules. See S10 Fig for compound **6–12** induced phenotypes.

Based on target prediction, we selected microtubules (α and β-tubulin) as our target for *in-vitro* validation. To test CSNAP’s prediction that 51 of the 212 mitotic compounds were targeting microtubules, we re-acquired all 212 compounds and tested their ability to perturb microtubule polymerization (stabilize or destabilize microtubules) in an *in-vitro* microtubule polymerization assay at 50μM concentration (Fig. 5A). The end-point absorbance (dOD) was used to quantify the degree of microtubule polymerization and was converted to percent fold change (F) relative to DMSO drug vehicle (0%), as previously described (Fig. 5A and S1 Text) [63]. Of the 51 compounds predicted to be targeting microtubules, 36 had more than 20% fold change in microtubule polymerization and 14 had no measurable effect (S5B Fig). Thus CSNAP was able to predict the targets of this set with > 70% accuracy. In addition, *in-vitro* testing led to the discovery of 96 additional compounds for a total of 132 anti-tubulin agents, including structurally diverse compounds covering ~54 novel chemotypes not discovered in previous chemical screens (S3 Table).

### Relating network connectivity to consensus drug mechanism

Since CSNAP was able to cluster compounds into sub-networks with respect to target specificities, we asked if ligands within the same chemotypic cluster shared a consensus drug-target binding mechanism, as shape complementarity between receptor surface and ligand geometry is essential for inducing a specific cellular phenotype. To test this, we mapped the tubulin polymerization activity onto the mitotic chemical similarity network. Overall, compounds with similar drug mechanisms, e.g. tubulin polymerization or depolymerization were clustered in close proximity within the CSN (S5A Fig). However, a few compounds with opposing mechanisms of action were clustered within the same sub-network. This was expected as chemical similarity may not always correlate with compound bioactivity [12]. Here, we investigated a chemical similarity sub-network consisting of 7 novel anti-tubulin ligands based on a phenyl-sulfanyl-thiazol-acetamide scaffold (Fig. 5B and S9B). Notably, all the connected ligands within the sub-network shared a similar microtubule destabilization effect. By conducting SAR analysis on the network, we noticed that the addition of hydrophobic groups to the northern and eastern parts of the ligand enhanced microtubule depolymerization (Fig. 5B and S1 Text). Consistently, a similar SAR trend was observed by evaluating each compound’s potency (EC_50_) in HeLa cells with regards to their ability to arrest cells in G2/M-phase and induce cell death. This identified compound 8 (EC_50:G2/M_ = 33 nM; EC_50: cell death_ = 60 nM) as the most potent compound in the series (S10 Fig and S1 Text).

To provide a structural explanation for this SAR, we observed that compound **6** shared a common structural feature (tri-methoxyphenyl ring) with the microtubule depolymerizer colchicine, suggesting that compounds **6–12**, within the sub-network may share a common colchicine-like binding mechanism (Fig. 5C) [53]. To test this hypothesis, we performed a structural alignment of compound **6** with colchicine and docked the aligned conformations onto the ligand-bound tubulin crystal structure (PDB: 1SA0) (Fig. 5C). Surprisingly, the predicted binding modes of the two molecules were conserved despite low structural similarity. As further validation of this binding mode, the same binding conformation was also recovered from the top poses by re-docking compound **6** into the colchicine binding site of an apo beta tubulin structure (chain B, PDB: 1FFX), giving a score of-10.82 (London dG) based on free energy binding of the ligand to the receptor site points. The docked structure revealed a consensus pharmacophore between the two aligned ligands including the 2 and 10-methoxy groups and a 9-keto group that interacted with Cys 241 of beta tubulin and Val 181 of alpha tubulin respectively, which had been previously reported (Fig. 5D) [52,64]. The docking of compounds **7–12** using the same approach also yielded similar binding interactions (S11 Fig). The discovery of this consensus-binding model for compounds **6–12** allowed us to link specific protein-ligand recognition features to compound network association and their SAR. For example, the receptor hydrophobicity map showed that the increased potency of compounds **7** and **8**, compared to **6**, could be attributed to the additional interaction of N-propyl group of compound **7** and the N-phenyl group of compound **8** within a sub-pocket enclosed between Leu 248 and Lys 352 of the colchicine-binding site, thus enhancing the protein-ligand interaction (Figs 5E and S11). To validate the binding of these compounds to the colchicine site, we used a mass spectrometry-based competition assay where compound **8** competed with colchicine for tubulin binding, similar to the positive control podophyllotoxin (colchicine site binder), and the negative control vincristine (vinca site binder) was unable to compete this interaction (Fig. 5F and S1 Text) [65]. To test if tubulin was the primary target, we treated HeLa cells with compounds **6–12** and analyzed their effects by IF microscopy. As expected, compounds **6–12** induced a microtubule depolymerization phenotype in HeLa cells (Figs 5G and S12). Thus, the structural binding analysis within a specific sub-network identified a relationship between network connectivity and consensus mechanism, likely due to shape complementarity between protein and ligands. Most importantly, this could be generalized as an effective strategy for structure-based target validation following CSNAP drug target prediction.

## Discussion

At the completion of cell-based chemical screening efforts researchers are faced with the daunting task of understanding drug mechanism of action and selecting lead compounds from a large number of structurally diverse hits to pursue further. To date, researchers have relied on experimental secondary screens, like multiparametric phenotypic profiling, to select a small number of compounds to validate, which is often costly to conduct and has reduced throughput [66]. On the other hand, computational approaches like simple chemical similarity searches do not capture the bioactivity correlation among the analyzed ligands, leading to prediction inconsistencies and low prediction accuracy. Our study demonstrated that CSNAP, a new computational target prediction methodology that uses chemical similarity networks coupled to a consensus-scoring scheme, improves the current state of the art in *in-silico* drug target identification. First, our benchmark study showed that CSNAP achieved a higher success rate than SEA, an approach based on sequential ligand similarity searches, at identifying pre-annotated drug targets from six major drug classes, especially for promiscuous ligands like CDK2 and ACE inhibitors. Since hit compounds from large chemical screens usually possess sub-optimal target specificity, CSNAP is particularly suitable for deconvolving these compounds compared to the existing approaches. Second, we applied CSNAP to predict and validate the drug targets of 212 mitotic compounds, whose drug binding mechanisms were previously unknown. Here, CSNAP was used in both a positive selection strategy to identify known compounds associated with three new categories of mitotic targets and in a negative selection strategy to identify novel chemotypes targeting microtubules, a major target in cancer drug discovery. Thus, we have demonstrated that CSNAP can achieve accurate large-scale drug target profiling of any compound set without relying on absolute chemical similarity or pre-conditioning from training sets.

However, CSNAP has several limitations. For instance, our tubulin polymerization assays indicated that around 30% of the tubulin targeting compounds were not predicted by CSNAP. This highlights the general limitation of any ligand-based approach, in that target annotation of the intended chemotype has to be deposited in the bioactivity database *a-priori*. Nevertheless, our structural studies of the novel microtubule depolymerizer compound **6**, whose pharmacophore aligned with the known microtubule targeting agent colchicine, suggests that a chemical similarity measure based on the three-dimensional structure of the compounds could potentially improve CSNAP’s prediction power. Likewise, the similarity between CSNAP networks and PPI networks provides further opportunities to apply different PPI network scoring schemes to improve CSNAP prediction [34]. For instance, neighbor counting functions could be readily expanded to consider second-order network neighbors, which has been shown to improve the prediction accuracy of PPI networks [67]. Finally, we showed that incorporating multiple databases, for example PubChem in conjunction with ChEMBL, improved the prediction range of the mitotic compounds by CSNAP. Thus, the simultaneous integration of multiple chemogenomic and bioinformatic knowledge databases can potentially aid the ability of CSNAP to predict the targets of any compound set.

In conclusion, we have developed a new network-based compound target identification method called CSNAP that can be used for large-scale profiling of hit compounds from chemical screens. To further extend the applicability of CSNAP for compound target prediction in a broad array of disciplines, we have made the CSNAP algorithm freely accessible as a CSNAP web server (http://services.mbi.ucla.edu/CSNAP/). The web server allows users to analyze up to 300 ligands in parallel, where each ligand can be processed in less than a minute on average (S13 Fig). We envision that CSNAP will be instrumental for deconvolving bioactive compounds from past and future cell-based studies relating to the discovery of antiproliferative agents and other processes related to cell division. More broadly, the flexibility of CSNAP to incorporate a wide variety of databases enables it to analyze any active compound set identified from any cell-based high throughput screen, thus expanding its utility across disciplines. Finally, CSNAP should expedite target identification and validation, while limiting costs associated with conventional target identification approaches.

## Materials and Methods

### Compounds

The benchmark validation sets were downloaded from the directory of useful decoys (DUD) VS 1.0 (http://dud.docking.org/jahn/). The mitotic compounds were retrieved from a vendor master compound SDfile. The ChEMBL reference compound databases were downloaded from the ChEMBL website (http://www.ebi.ac.uk/chembl/).

### *In-vitro* microtubule polymerization assays

A stock plate of the 212 mitotic compounds was prepared by transferring each drug in DMSO into a 384 well plate at a final concentration of 500 μM. Tubulin polymerization assays were conducted using HTS-Tubulin polymerization assay kit from Cytoskeleton Inc. To minimize pre-mature tubulin polymerization, 24 reactions were tested per run using multi-channel pipettes. Briefly, a 500 μM solution of each test compound and control compounds (Nocodazole and Taxol) were prepared in DMSO and subsequently diluted in ice-cold G-PEM buffer [80 mmol/L PIPES (pH 6.9), 2.0 mmol/L, MgCl_2_, 0.5 mmol/L EGTA, 1.0 mmol/L GTP] to a final concentration of 50 μM. Lyophilized bovine brain tubulin was resuspended in ice-cold G-PEM buffer to a final concentration of 4 mg/ml. Test compounds were added to each well (2μl/well) of a 384 well plate followed by the addition of tubulin (20μl/well). The reactions were assembled on ice to prevent tubulin pre-polymerization. The final concentration of test compounds was 50 μM in 0.5% DMSO. To measure tubulin polymerization kinetics, the plate was warmed to 37°C in a Tecan microplate reader (Tecan Group Ltd.) and read at 340 nm every minute for total of 1 hour.

### Competitive mass spectrometry binding assay

Colchicine (1.2 μM) was incubated with porcine brain tubulin (1.0 mg/mL) in incubation buffer [80 mM piperazine-N,N′-bis(2-ethanesulfonic acid) (PIPES), 2.0 mM magnesium chloride (MgCl2), 0.5 mM ethylene glycol tetra acetic acid (EGTA), pH 6.9] at 37°C for 1 hour. Test compounds (100 μM) were added to compete with the binding of colchicine to tubulin. After 1 h incubation, the filtrate was obtained using an ultrafiltration method (microconcentrator) (Microcon, Bedford, MA) with a molecular cut-off size of 30 kDa. The ability of the compounds of interest to inhibit the binding of colchicine was expressed as a percentage of control binding in the absence of any competitor. Each experiment was performed in triplicate.

### Cell culture

HeLa cells were grown in F12:DMEM 50:50 medium (GIBCO) with 10% FBS, 2 mM L-glutamine and antibiotics in 5% CO2 at 37°C.

### Immunofluorescence microscopy

Immunofluorescence was carried out essentially as described previously [68]. HeLa cells were treated with indicated compounds at their respective EC_90_ for 20 hours, fixed with 4% paraformaldehyde, permeabilized with 0.2% Triton X-100/PBS and co-stained for DNA (0.5 μg/ml Hoechst 33342) and tubulin (rat anti-tubulin primary antibodies and anti-rat Cy3 secondary antibodies). Images were captured with a Leica DMI6000 microscope at 63X magnification.

### Molecular modeling

The crystal structure of colchicine-bound tubulin was downloaded from the PDB database (PDB code: 1SA0) and the beta tubulin monomer with bound colchicine (chain D) was extracted from the protein model [69]. Compounds 6–12 were flexible aligned with colchicine within the colchicine-binding site using the “flexible alignment” protocol and default parameters (alignment mode: flexible, iteration limit: 200, failure limit: 20, energy cutoff: 15, stochastic conformation search), which gave a score for each alignment by quantifying the quality of internal strain and overlap of molecular features. Additionally, we realigned the colchicine structure with its crystal-derived conformation to ensure accuracy of the protocol. The aligned conformation of each compound was subsequently energy minimized within the colchicine-binding pocket using the LigX protocol. The re-docking of compound **6** into the colchicine-binding site was performed using the Dock protocol with default parameters (placement: triangle matcher, score: London dG, retained conformations: 30). The molecular modeling was performed using the MOE software version 2009.

### Statistical analysis

The mean and standard deviations of DMSO and Taxol controls for the *in-vitro* tubulin polymerization assays were calculated and used to scale the compound OD readout between different runs to normalize the heterogeneity of the reaction. All the statistical analysis for *in-vitro* tubulin polymerization assays was performed using Microsoft Excel.

### Software

The CSNAP program is written in shell scripting language and Perl programming language on Ubuntu 12.10 Linux operating system. The program is dependent on the following external programs/scripts including OBABEL version 2.3.1 and NCI SDF toolkit version 1.2. Additionally, the R statistical package and Cytoscape version 2.8.2 were applied for visualizing and analyzing heat maps and networks respectively. See Supporting Information for program description and tutorials. The CSNAP program is freely accessible from the CSNAP web server (http://services.mbi.ucla.edu/CSNAP/).

### Supporting information

Supporting Information includes Supporting Materials and Methods, thirteen figures, four tables, two supporting files, and supporting tutorials and can be found with this article online.

## Supporting Information

S1 Fig CSNAP Analysis of benchmark compounds based on an alternative chemical similarity search criteria, related to Fig. 2. Performing CSNAP analysis of the benchmark compounds using a lower Tc threshold (Tc cutoff = 0.85 and Z-score cutoff = 2.5, ChEMBL version 16) in comparison to using a higher threshold criteria (Z-score cutoff = 2.5, Tc-score cutoff = 1, ChEMBL version 16) shown in Fig. 2A, leads to a substantial increase in network density (number of nodes) but does not significantly change the number of chemical similarity clusters.
(PDF)

S2 Fig CSNAP target identification and LTIF analysis of the benchmark compound sets, related to Fig. 2. The benchmark compounds comprised of six drug classes (CDK2, ACE, HMGA, PARP, HIVRT, and HSP90) were combined and analyzed by CSNAP followed by LTIF analysis. The target spectrum represented by the sum of S-scores of each predicted target, were used to identify the major targets from the top peaks. The results showed that all of the six labeled drug targets and predicted off-targets were identified from the target spectrum.
(PDF)

S3 Fig Integration of CSNAP analysis with the MitoCheck knowledge database for mitotic target identification, related to Fig. 4. Workflow for integrating CSNAP analysis with the knowledge database MitoCheck (maintains data on the mitotic phenotypes observed upon siRNA gene expression knockdown for almost all human genes) for mitotic drug target identification. 212 mitotic compounds with unknown drug targets from chemical screens were analyzed by the CSNAP program and 116 predicted target IDs were retrieved. These targets were analyzed by LTIF analysis with a predefined cutoff (∑ S-score >10), from which we identified 4 broad categories of putative targets (20 UniProt target IDs) from the top peaks of the target spectrum (See S4 and S5 Figs for query results).
(PDF)

S4 Fig Target and off-target prediction of the mitotic compounds, related to Fig. 4. The mitotic compounds with four predicted mitotic targets by CSNAP analysis were analyzed by LTIF to determine their off-target effects. The LTIF analysis of SCD and ABL1 reveals several compounds targeting both SCD and ABL1 in each target category.
(PDF)

S5 Fig Identification of novel tubulin-targeting agents by CSNAP analysis, related to Fig. 4 and 5. (A) 212 antimitotic compounds clustered into 85 distinct chemical similarity sub-networks of which 23 clusters contained annotated anti-tubulin agents (green); additionally 54 novel tubulin-targeting chemotypes (yellow) were identified from *in-vitro* tubulin polymerization assays. (B) The first-order neighbors of the anti-tubulin compounds were extracted from the chemical similarity sub-network, resulting in 24 similarity clusters. Of the 51 compounds predicted to be targeting microtubules, 36 compounds (71%) had more than 20% fold change in *in-vitro* tubulin polymerization assay and 14 had no measurable effect.
(PDF)

S6 Fig Integration of CSNAP with knowledge databases for mitotic target prediction related to Fig. 4. CSNAP analysis of 212 mitotic compounds predicted 20 mitotic targets. The MitoCheck database confirmed 14 targets within 4 broad categories: SCD, ABL1, PTPN, and TUBB, whose depletion induced a mitotic defect phenotype and are potential targets for these compounds. The color intensity of the band correlates with the number of successful replicates for the target phenotype by siRNA knockdown.
(PDF)

S7 Fig Mitotic phenotypes of target subtypes, related to Fig. 4. All subtypes within each of the 4 predicted target categories (SCD, PTPN, ABL1 and TUBB) were searched within the MitoCheck database. Note that all four target categories display diverse mitotic phenotypes by siRNA knockdown.
(PDF)

S8 Fig Phenotypic analysis of SCD, ABL1, PTPN and TUBB compound classes, related to Fig. 4. (A-F) Immunofluorescence of HeLa cells treated with control DMSO or indicated compounds (**1–5**) for 20 hours. Cells were fixed with paraformaldehyde, permeabilized and stained for DNA and tubulin. Legend describes the different types of observed phenotypes corresponding to the reported MitoCheck phenotypes for siRNA-mediated knockdown of predicted compound targets. Scale = 5 μm.
(PDF)

S9 Fig Identification of a compound sub-network with a consensus tubulin destabilizing effect, related to Fig. 5. (A) Mapping of tubulin polymerization activity onto the mitotic compound set CSN identified a compound sub-network with a consensus tubulin destabilization effect. (B) Tubulin polymerization kinetics for 7 novel tubulin destabilizers (**6–12**), based on a phenyl-sulfanyl-thiazol-acetamide scaffold, using an *in-vitro* tubulin polymerization assay. Note that all compounds inhibited tubulin polymerization compared to the DMSO control and tubulin stabilizer Taxol control.
(PDF)

S10 Fig Determination of compound potency in cell culture, related to Fig. 5. (A) For cell viability assays, HeLa cells were treated with increasing concentrations (20-point titration 0–100 μM) of indicated compounds (**6–12**) for 20 hours and the percentage of cells arrested in G2/M was quantified. (B) For cell cycle arrest assays, cells were treated with compounds for 72 hours and the extent of cell death was quantified. The EC_50s_ were calculated using the CDD (Collaborative Drug Discovery) software. See Extended Experimental Procedures for complete details.
(PDF)

S11 Fig Molecular modeling of structural binding mechanism for compounds 6–12, related to Fig. 5. Structural alignment of compounds **6–12** within the colchicine-binding pocket of the colchicine-tubulin crystal structure (PDB: 1SA0) using the MOE FlexAlign protocol followed by an energy minimization procedure to simulate the “induced-fit” effect. All protein-ligand complexes showed similar binding modes and were consistent with the SAR analysis.
(PDF)

S12 Fig Phenotypic analysis of microtubule destabilizing compounds 6–12, related to Fig. 5. Immunofluorescence microscopy of HeLa cells treated with control DMSO, Taxol, colchicine, or the indicated compounds (**6–12**) for 20 hours. Cells were fixed with paraformaldehyde, permeabilized, and stained for DNA (Hoechst 33342) and tubulin (primary rat anti-tubulin antibodies and secondary anti-rat Cy3 antibodies). Note that all compounds showed a microtubule destabilization effect similar to colchicine-treatment. Scale = 5 μm.
(PDF)

S13 Fig CSNAP web performance benchmark. To evaluate CSNAP Web performance, an increasing number of ligands (6–96) from the benchmark set containing six drug classes (CDK2, ACE, HMGA, PARP, HIVRT, and HSP90) were input as queries and the total processing time (minutes) was measured using default chemical search parameters. Each input compound set was selected randomly in triplicate from each drug class and the average total processing time for each number of compound set was computed. Regression analysis (y = 0.2951x+0.8667, R^2^ = 0.9342) showed a linear relationship between the processing time and the number of input ligands where each ligand was processed in less than a minute on average.
(PDF)

S1 Table Benchmark compound structure and methods comparison data, related to Fig. 3. Complete list of benchmark compound data in SMILES notation and their target prediction results analyzed by CSNAP and SEA approaches respectively. The SMILES data were converted from the original benchmark compound SD file downloaded from the DUD LIB VS 1.0 set (http://dud.docking.org/). The top hits ranked by each respective measure (S-score or E-value) were recorded (CSNAP top hit or SEA top hit). If the labeled target did not match the top hit, the rank of labeled targets were identified as rank (labeled) and the corresponding scores were recorded as S-score (label) or E-val (label) respectively.
(XLS)

S2 Table Small molecule screening data, related for Fig. 4. Complete description of HTS assay, compound library, screening conditions and post HTS analyses.
(PDF)

S3 Table List of 212 mitotic compounds and results of *in-vitro* tubulin polymerization assays, related to Fig. 5. The effect of the 212 mitotic compounds on microtubule assembly was analyzed using an *in-vitro* tubulin polymerization assay. The end-point absorbance based on change in OD (dOD) was used to quantify the degree of microtubule polymerization and was converted to percentage fold change relative to DMSO (0%). The percentage fold change is listed for each compound.
(XLS)

S4 Table Target identification for compounds 1–5, related to Fig. 4. Five compounds (one from each of the five predicted target chemical similarity sub-networks) were selected for phenotypic analysis including compound 1 from the SCD sub-cluster (cluster 6), compound 2 that overlapped with both SCD and ABL1 sub-clusters (cluster 6) and compound 3 from the ABL1 sub-cluster (cluster 6). Additionally, compound 4 and compound 5, were retrieved from the PTPN cluster (cluster 3) and the TUBB cluster (cluster 4) respectively. Note that the reference ChEMBL compounds are in gray, the mitotic compounds are in red and the selected compounds are in yellow.
(PDF)

S1 Text Supporting tutorials, supporting materials and methods, supporting references. (PDF)

S1 File CSNAP analysis results of benchmark sets (benchmark.cys) (Tc-cutoff = 0.85) for visualization using Cytoscape. (CYS)

S2 File CSNAP analysis results of mitotic sets (mitotic.cys) (Tc-cutoff = 0.85) for visualization using Cytoscape. (CYS)
