# Supplementary figures and images for: Large-Scale Chemical Similarity Networks for Target Profiling of Compounds Identified in Cell-Based Chemical Screens
Source: PLoS Comput Biol. 2015 Mar 31;11(3):e1004153. doi: 10.1371/journal.pcbi.1004153 (PMC4380459; doi:10.1371/journal.pcbi.1004153)

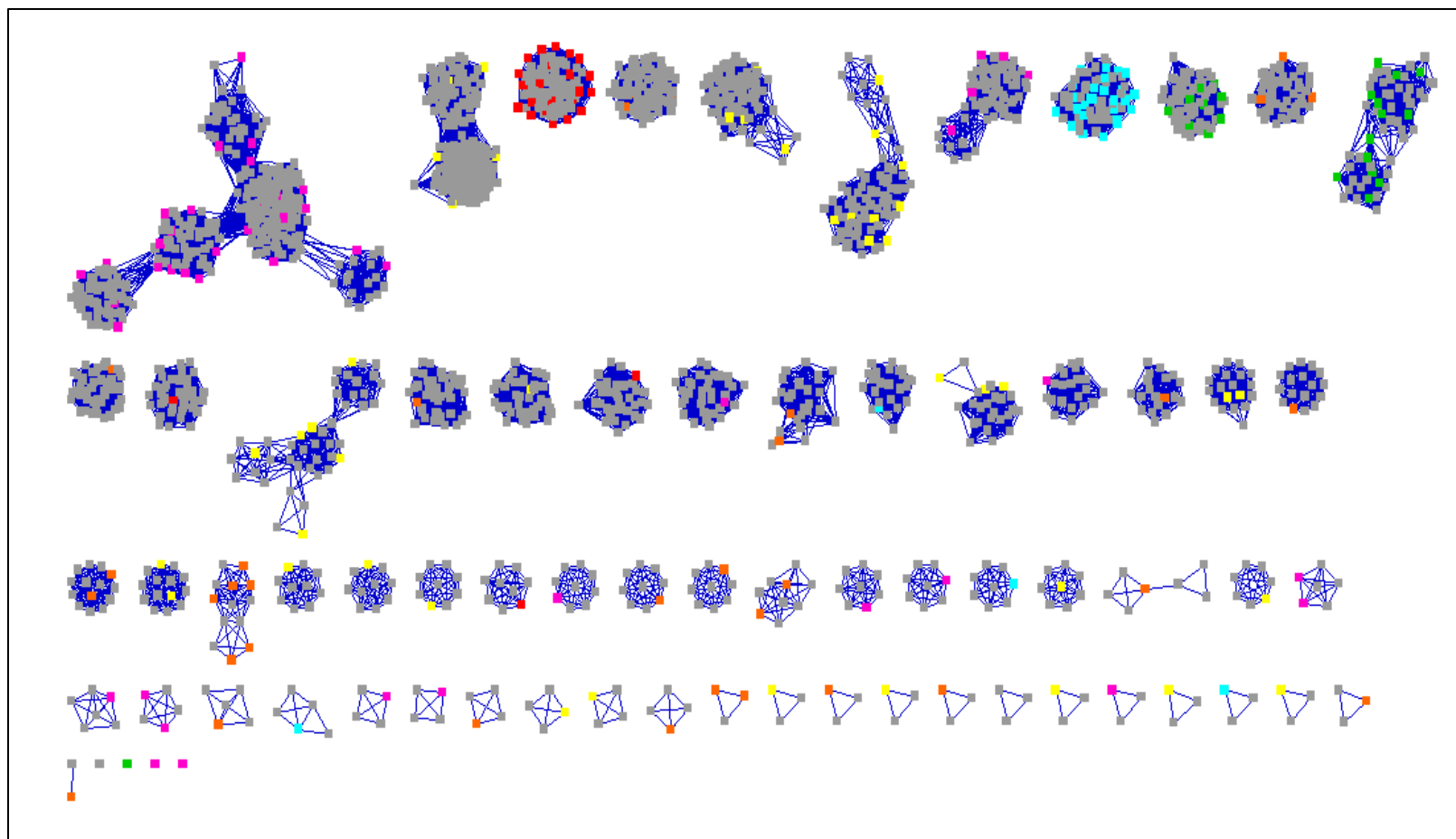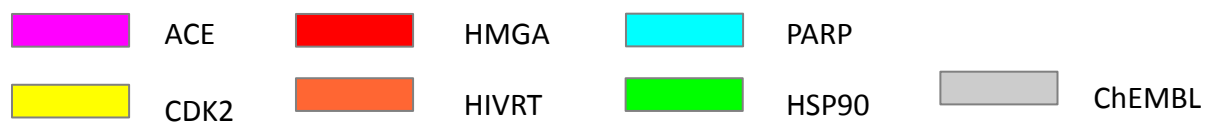

Supplement: S1 Fig — Performing CSNAP analysis of the benchmark compounds using a lower Tc threshold (Tc cutoff = 0.85 and Z-score cutoff = 2.5, ChEMBL version 16) in comparison to using a higher threshold criteria (Z-score cutoff = 2.5, Tc-score cutoff = 1, ChEMBL version 16) shown in Fig. 2A, leads to a substantial increase in network density (number of nodes) but does not significantly change the number of chemical similarity clusters. (PDF) [file pcbi.1004153.s001.pdf]

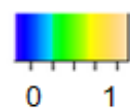

206 Compound ID

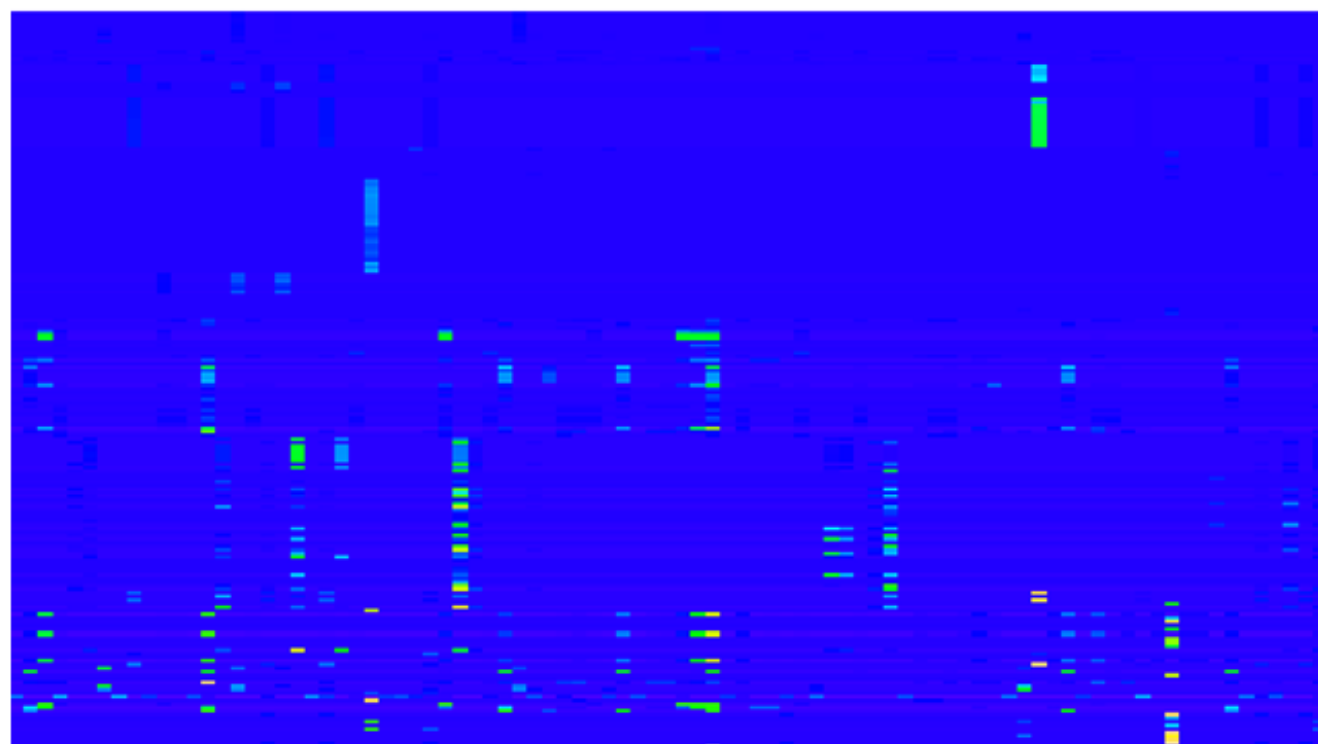

Uniprot ID

$\Sigma$ s-score

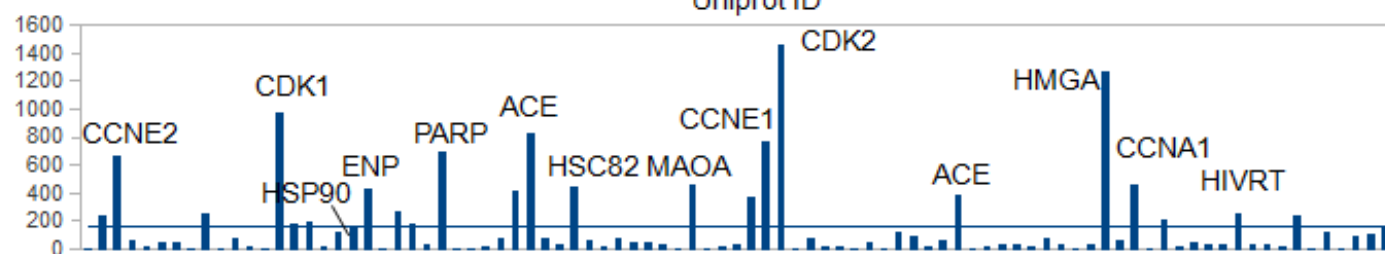

Supplement: S2 Fig — The benchmark compounds comprised of six drug classes (CDK2, ACE, HMGA, PARP, HIVRT, and HSP90) were combined and analyzed by CSNAP followed by LTIF analysis. The target spectrum represented by the sum of S-scores of each predicted target, were used to identify the major targets from the top peaks. The results showed that all of the six labeled drug targets and predicted off-targets were identified from the target spectrum. (PDF) [file pcbi.1004153.s002.pdf]

## TUBB

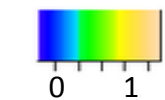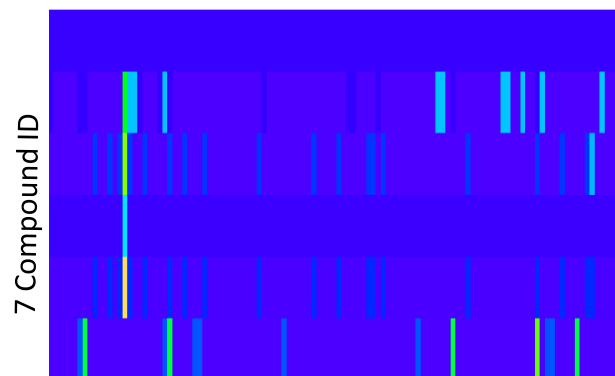

$\Sigma$ S-score

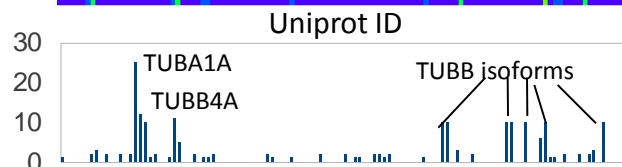

## SCD

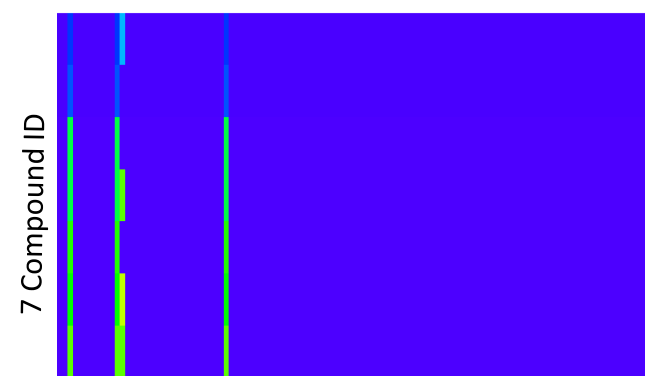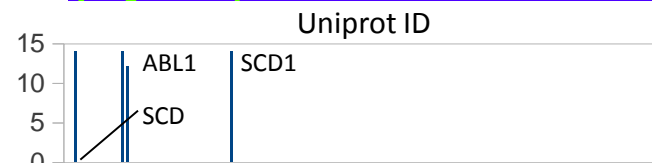

## PTPN

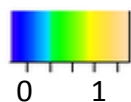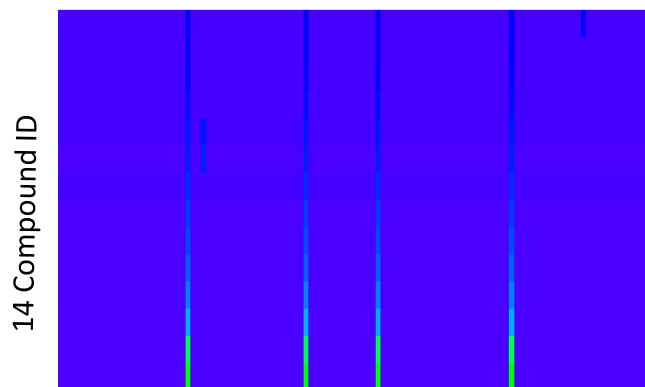

$\Sigma$ S-score

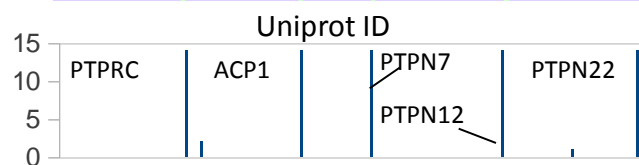

## ABL1

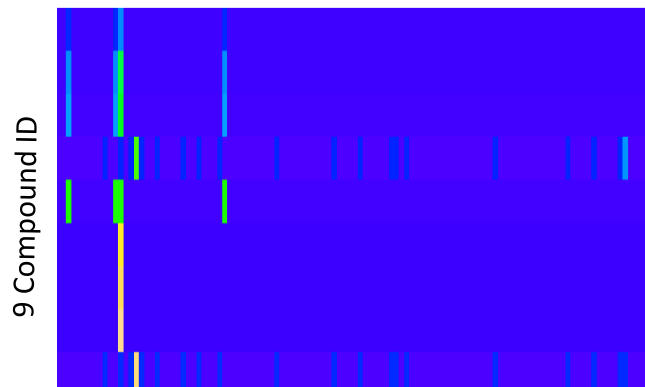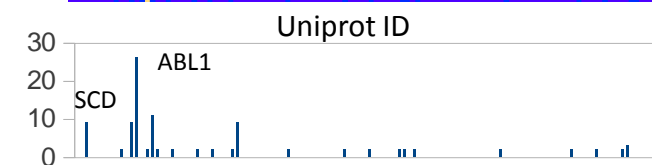

Supplement: S4 Fig — The mitotic compounds with four predicted mitotic targets by CSNAP analysis were analyzed by LTIF to determine their off-target effects. The LTIF analysis of SCD and ABL1 reveals several compounds targeting both SCD and ABL1 in each target category. (PDF) [file pcbi.1004153.s004.pdf]

**A**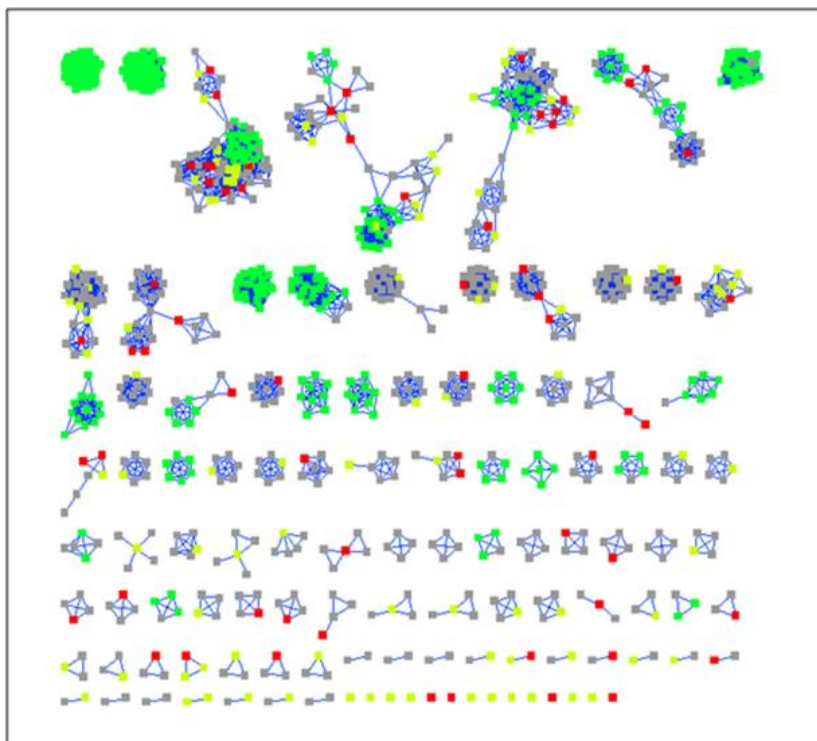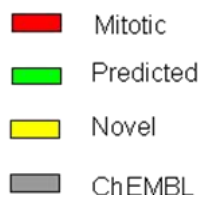**B**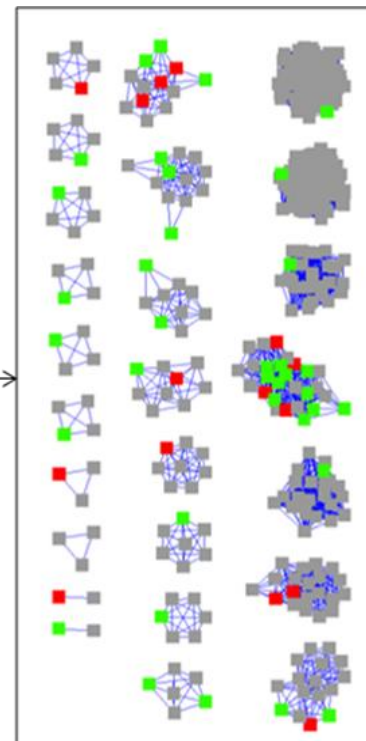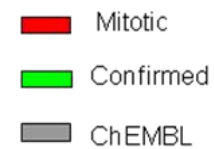

Supplement: S5 Fig — (A) 212 antimitotic compounds clustered into 85 distinct chemical similarity sub-networks of which 23 clusters contained annotated anti-tubulin agents (green); additionally 54 novel tubulin-targeting chemotypes (yellow) were identified from in-vitro tubulin polymerization assays. (B) The first-order neighbors of the anti-tubulin compounds were extracted from the chemical similarity sub-network, resulting in 24 similarity clusters. Of the 51 compounds predicted to be targeting microtubules, 36 compounds (71%) had more than 20% fold change in in-vitro tubulin polymerization assay and 14 had no measurable effect. (PDF) [file pcbi.1004153.s005.pdf]

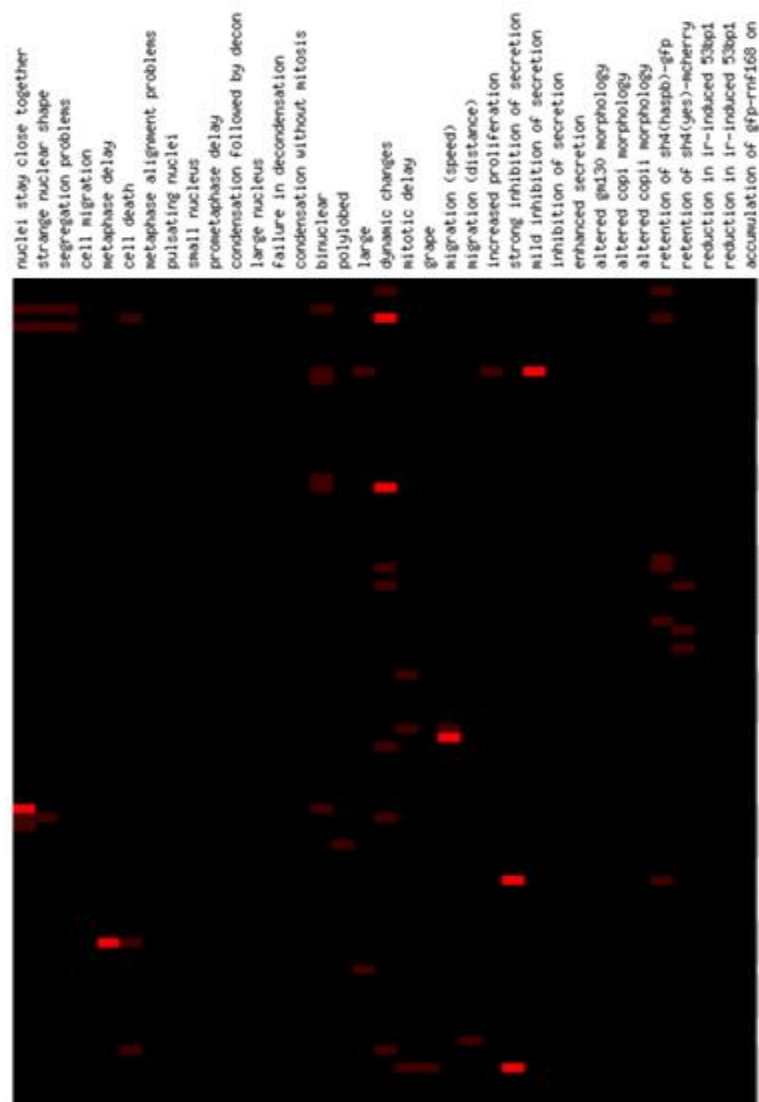

SCD  
 FADS2  
 ABL1  
 TUBA1A  
 TUBB  
 FYN  
 PTPRC  
 MAPT  
 ACP1  
 PTPN7  
 TUBA4A  
 PTPN12  
 TUBB3  
 TUBA3D  
 TUBA8A  
 TUBB1  
 PTPN22

Supplement: S6 Fig — CSNAP analysis of 212 mitotic compounds predicted 20 mitotic targets. The MitoCheck database confirmed 14 targets within 4 broad categories: SCD, ABL1, PTPN, and TUBB, whose depletion induced a mitotic defect phenotype and are potential targets for these compounds. The color intensity of the band correlates with the number of successful replicates for the target phenotype by siRNA knockdown. (PDF) [file pcbi.1004153.s006.pdf]

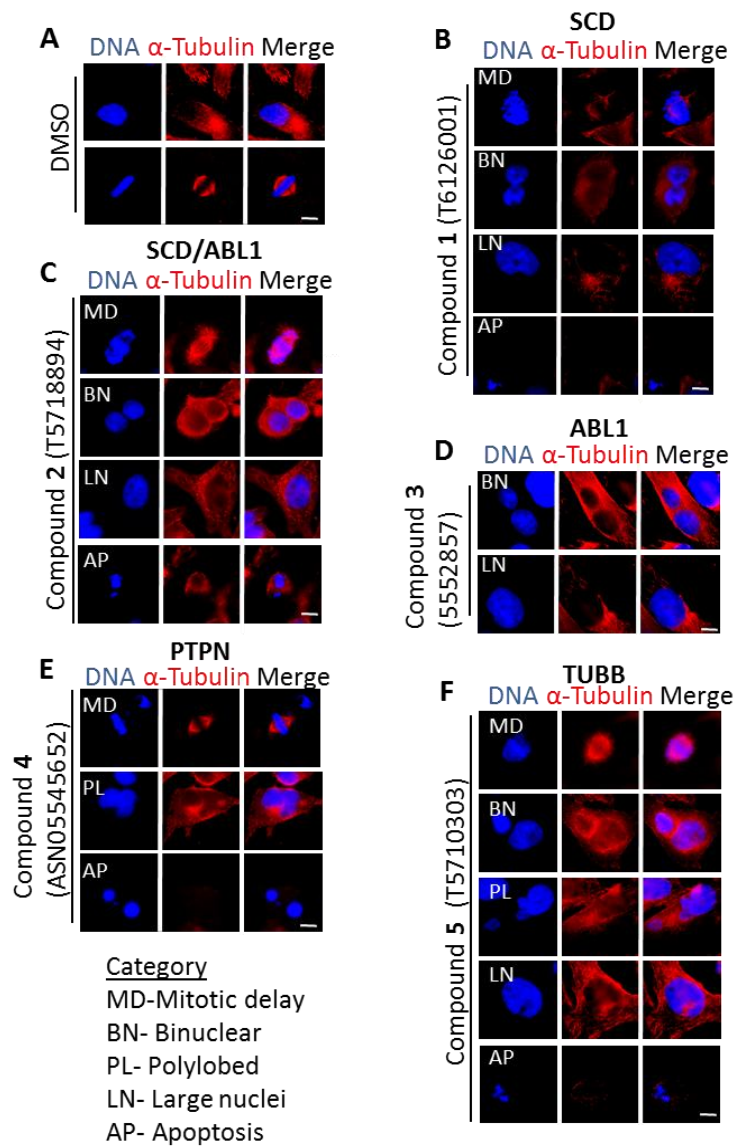

Supplement: S8 Fig — (A-F) Immunofluorescence of HeLa cells treated with control DMSO or indicated compounds (1–5) for 20 hours. Cells were fixed with paraformaldehyde, permeabilized and stained for DNA and tubulin. Legend describes the different types of observed phenotypes corresponding to the reported MitoCheck phenotypes for siRNA-mediated knockdown of predicted compound targets. Scale = 5 μm. (PDF) [file pcbi.1004153.s008.pdf]

**A**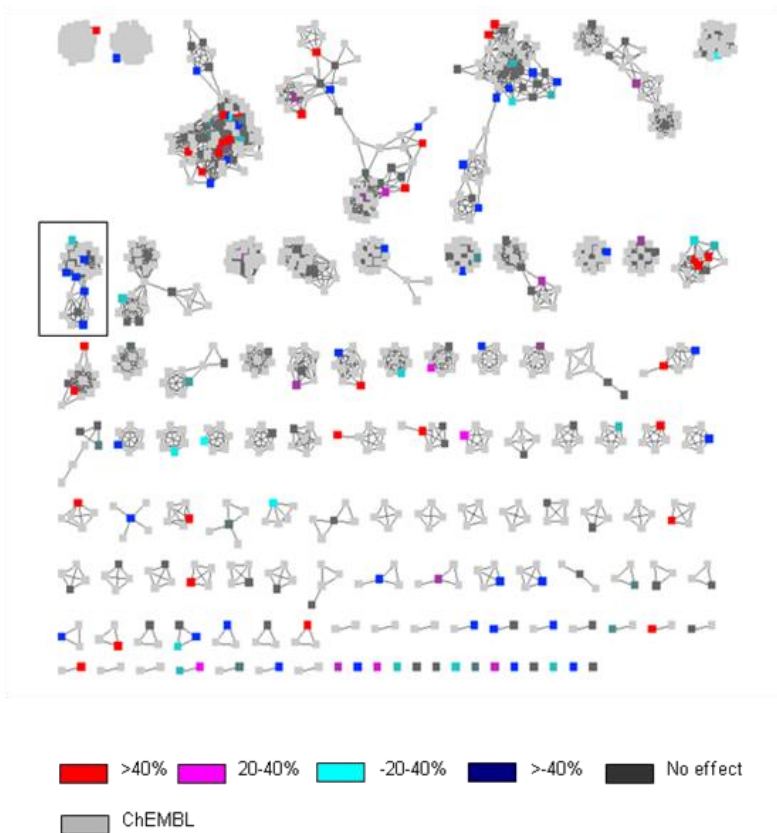**B**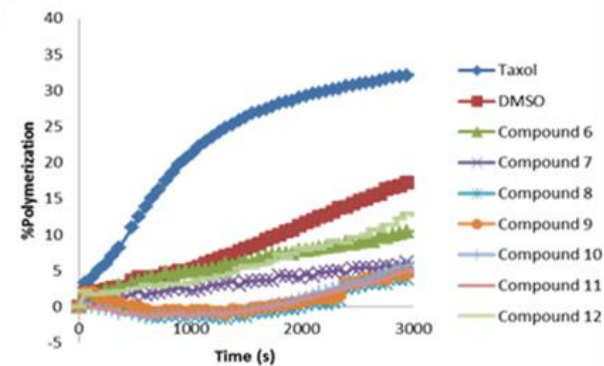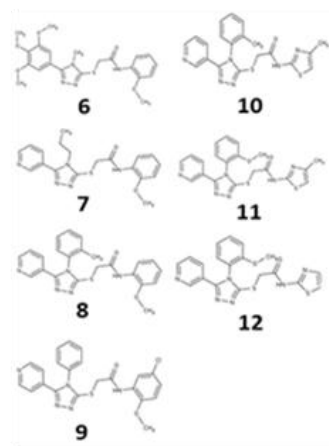

Supplement: S9 Fig — (A) Mapping of tubulin polymerization activity onto the mitotic compound set CSN identified a compound sub-network with a consensus tubulin destabilization effect. (B) Tubulin polymerization kinetics for 7 novel tubulin destabilizers (6–12), based on a phenyl-sulfanyl-thiazol-acetamide scaffold, using an in-vitro tubulin polymerization assay. Note that all compounds inhibited tubulin polymerization compared to the DMSO control and tubulin stabilizer Taxol control. (PDF) [file pcbi.1004153.s009.pdf]

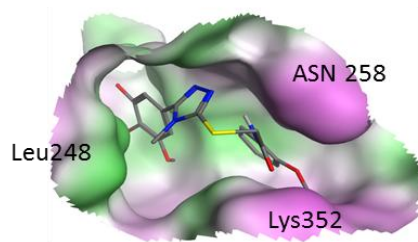

Compound 6

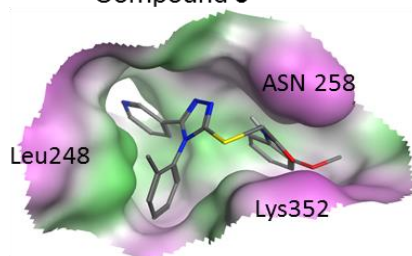

Compound 8

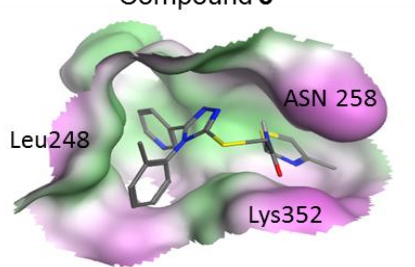

Compound 10

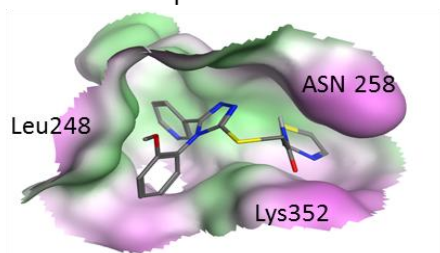

Compound 12

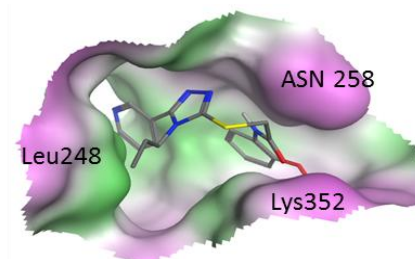

Compound 7

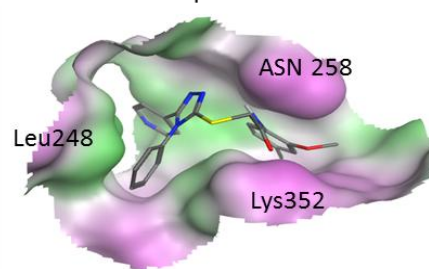

Compound 9

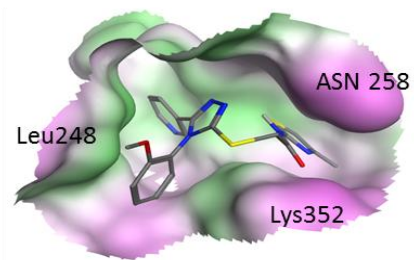

Compound 11

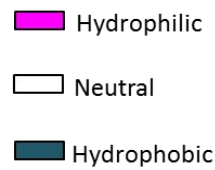

Supplement: S11 Fig — Structural alignment of compounds 6–12 within the colchicine-binding pocket of the colchicine-tubulin crystal structure (PDB: 1SA0) using the MOE FlexAlign protocol followed by an energy minimization procedure to simulate the “induced-fit” effect. All protein-ligand complexes showed similar binding modes and were consistent with the SAR analysis. (PDF) [file pcbi.1004153.s011.pdf]

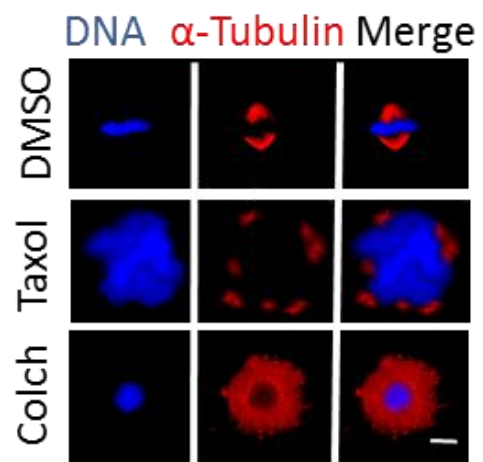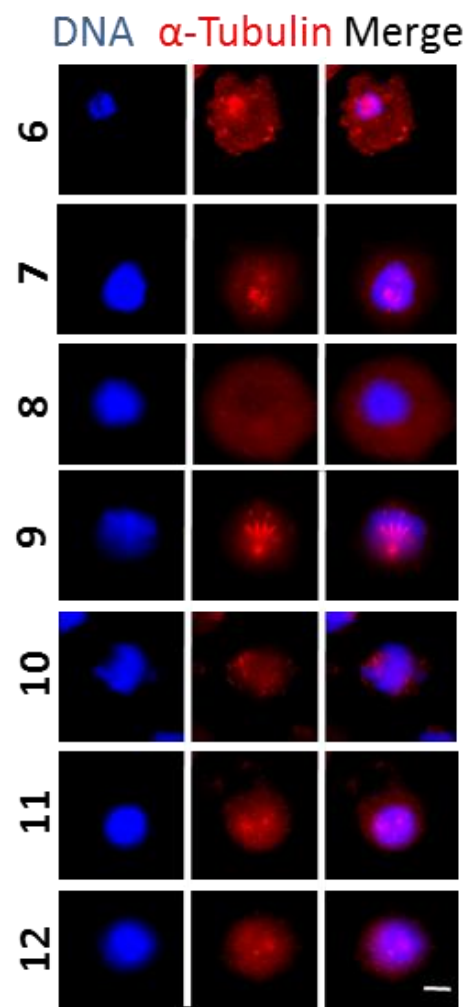

Supplement: S12 Fig — Immunofluorescence microscopy of HeLa cells treated with control DMSO, Taxol, colchicine, or the indicated compounds (6–12) for 20 hours. Cells were fixed with paraformaldehyde, permeabilized, and stained for DNA (Hoechst 33342) and tubulin (primary rat anti-tubulin antibodies and secondary anti-rat Cy3 antibodies). Note that all compounds showed a microtubule destabilization effect similar to colchicine-treatment. Scale = 5 μm. (PDF) [file pcbi.1004153.s012.pdf]

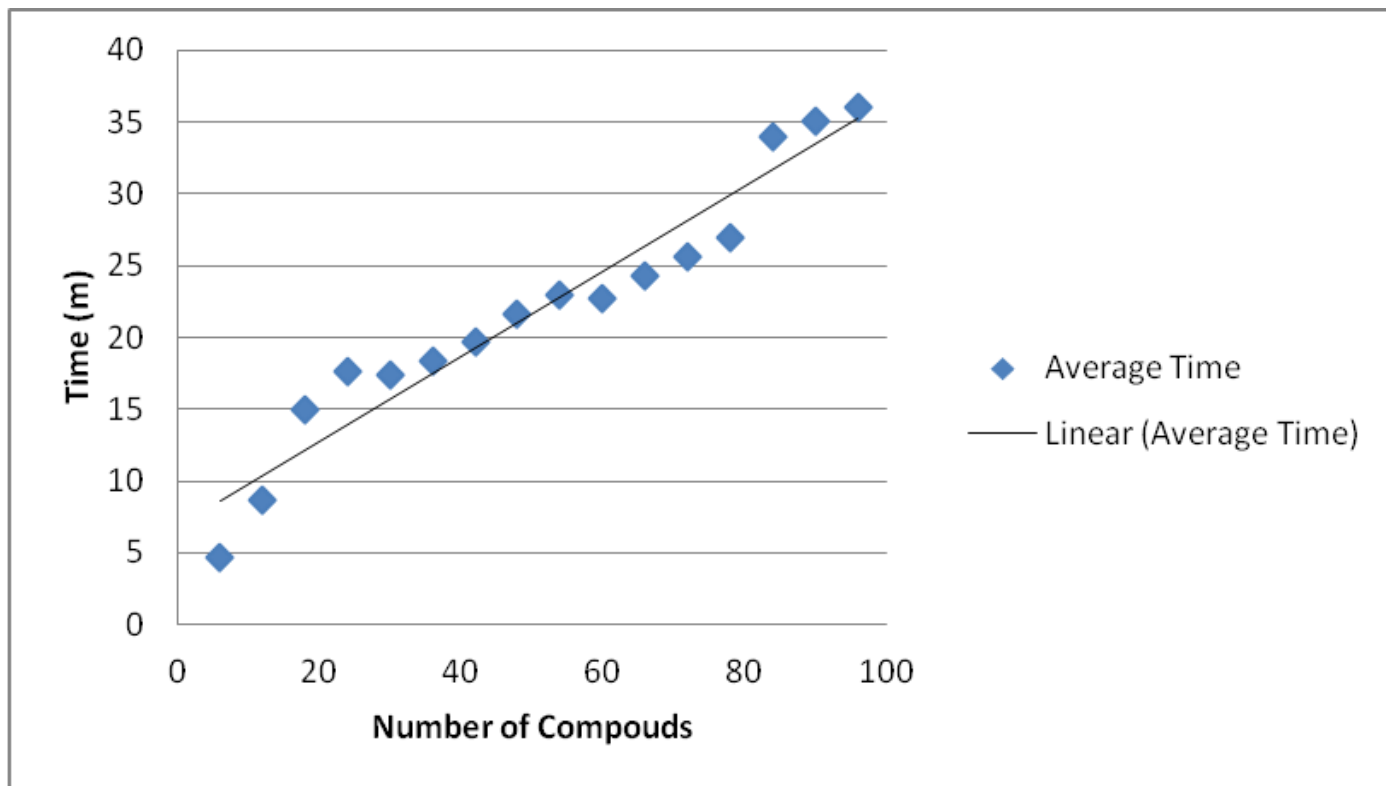

Supplement: S13 Fig — To evaluate CSNAP Web performance, an increasing number of ligands (6–96) from the benchmark set containing six drug classes (CDK2, ACE, HMGA, PARP, HIVRT, and HSP90) were input as queries and the total processing time (minutes) was measured using default chemical search parameters. Each input compound set was selected randomly in triplicate from each drug class and the average total processing time for each number of compound set was computed. Regression analysis (y = 0.2951x+0.8667, R2 = 0.9342) showed a linear relationship between the processing time and the number of input ligands where each ligand was processed in less than a minute on average. (PDF) [file pcbi.1004153.s013.pdf]
