# Supplementary material for: Large-Scale Chemical Similarity Networks for Target Profiling of Compounds Identified in Cell-Based Chemical Screens
Source: PLoS Comput Biol. 2015 Mar 31;11(3):e1004153. doi: 10.1371/journal.pcbi.1004153 (PMC4380459; doi:10.1371/journal.pcbi.1004153)
Supplement: S3 Fig — Workflow for integrating CSNAP analysis with the knowledge database MitoCheck (maintains data on the mitotic phenotypes observed upon siRNA gene expression knockdown for almost all human genes) for mitotic drug target identification. 212 mitotic compounds with unknown drug targets from chemical screens were analyzed by the CSNAP program and 116 predicted target IDs were retrieved. These targets were analyzed by LTIF analysis with a predefined cutoff (∑ S-score >10), from which we identified 4 broad categories of putative targets (20 UniProt target IDs) from the top peaks of the target spectrum (See S4 and S5 Figs for query results). (PDF) [file pcbi.1004153.s003.pdf]

212 mitotic compounds

↓ *CSNAP target prediction*

116 target IDs

↓ *LTIF analysis (∑S-score>10)*

20 target IDs

↓ *Mitocheck database search*

14 target induced mitotic phenotypes

↓ *Comparison of compound-Induced phenotype*

Compound deconvolution
