# Supplementary material for: Large-Scale Chemical Similarity Networks for Target Profiling of Compounds Identified in Cell-Based Chemical Screens
Source: PLoS Comput Biol. 2015 Mar 31;11(3):e1004153. doi: 10.1371/journal.pcbi.1004153 (PMC4380459; doi:10.1371/journal.pcbi.1004153)
Supplement: S7 Fig — All subtypes within each of the 4 predicted target categories (SCD, PTPN, ABL1 and TUBB) were searched within the MitoCheck database. Note that all four target categories display diverse mitotic phenotypes by siRNA knockdown. (PDF) [file pcbi.1004153.s007.pdf]

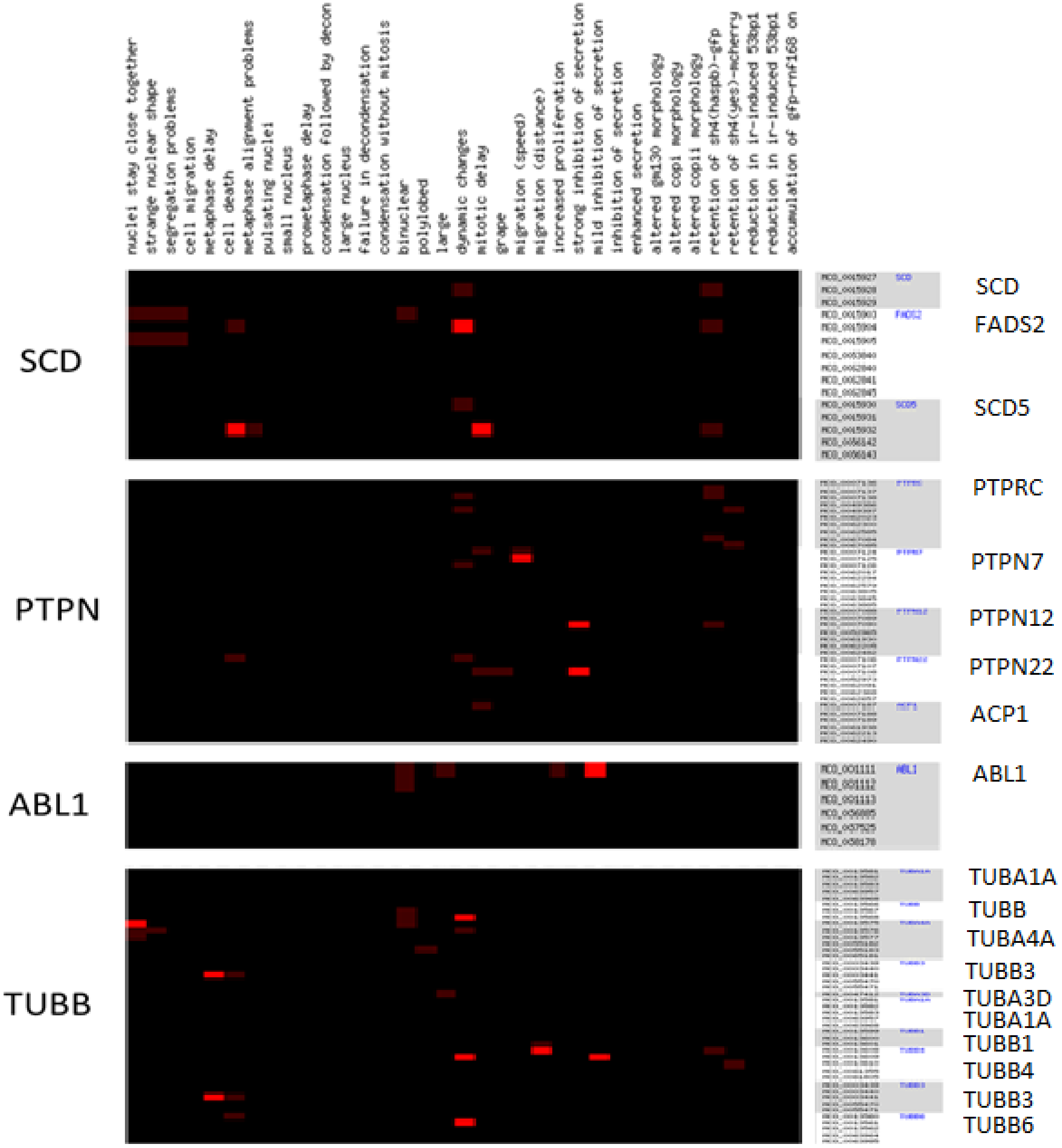
nuclei stay close together
strange nuclear shape
segregation problems
cell migration
metaphase delay
cell death
metaphase alignment problems
pulsating nuclei
small nucleus
prometaphase delay
condensation followed by decon
large nucleus
failure in decondensation
condensation without mitosis
binuclear
polylobed
large
dynamic changes
mitotic delay
grape
migration (speed)
migration (distance)
increased proliferation
strong inhibition of secretion
mild inhibition of secretion
inhibition of secretion
enhanced secretion
altered gm130 morphology
altered copi morphology
altered copii morphology
retention of sh4(haspb)-gfp
retention of sh4(yes)-mcherry
reduction in ir-induced 53bp1
reduction in ir-induced 53bp1
accumulation of gfp-rnf168 on
SCD
PTPN
ABL1
TUBB
SCD
FADS2
SCD5
PTPRC
PTPN7
PTPN12
PTPN22
ACP1
ABL1
TUBA1A
TUBB
TUBA4A
TUBB3
TUBA3D
TUBA1A
TUBB1
TUBB4
TUBB3
TUBB6
