# Supplementary material for: Large-Scale Chemical Similarity Networks for Target Profiling of Compounds Identified in Cell-Based Chemical Screens
Source: PLoS Comput Biol. 2015 Mar 31;11(3):e1004153. doi: 10.1371/journal.pcbi.1004153 (PMC4380459; doi:10.1371/journal.pcbi.1004153)
Supplement: S10 Fig — (A) For cell viability assays, HeLa cells were treated with increasing concentrations (20-point titration 0–100 μM) of indicated compounds (6–12) for 20 hours and the percentage of cells arrested in G2/M was quantified. (B) For cell cycle arrest assays, cells were treated with compounds for 72 hours and the extent of cell death was quantified. The EC50s were calculated using the CDD (Collaborative Drug Discovery) software. See Extended Experimental Procedures for complete details. (PDF) [file pcbi.1004153.s010.pdf]

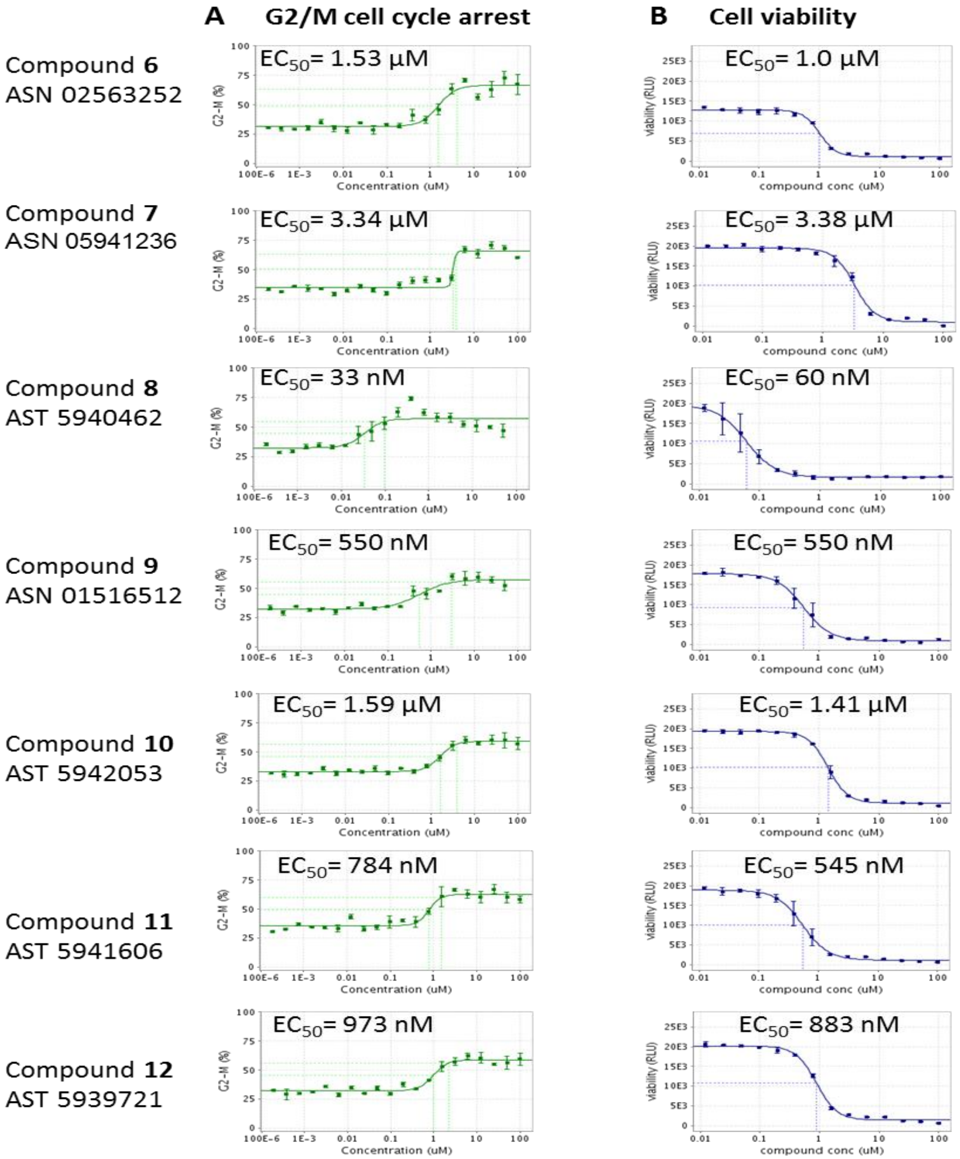

A G2/M cell cycle arrest
B Cell viability
Compound 6
ASN 02563252
EC50= 1.53 µM
EC50= 1.0 µM
Compound 7
ASN 05941236
EC50= 3.34 µM
EC50= 3.38 µM
Compound 8
AST 5940462
EC50= 33 nM
EC50= 60 nM
Compound 9
ASN 01516512
EC50= 550 nM
EC50= 550 nM
Compound 10
AST 5942053
EC50= 1.59 µM
EC50= 1.41 µM
Compound 11
AST 5941606
EC50= 784 nM
EC50= 545 nM
Compound 12
AST 5939721
EC50= 973 nM
EC50= 883 nM
G2-M (%)
Concentration (uM)
viability (RLU)
compound conc (uM)
