# Supplementary material for: Large-Scale Chemical Similarity Networks for Target Profiling of Compounds Identified in Cell-Based Chemical Screens
Source: PLoS Comput Biol. 2015 Mar 31;11(3):e1004153. doi: 10.1371/journal.pcbi.1004153 (PMC4380459; doi:10.1371/journal.pcbi.1004153)
Supplement: S2 Table — Complete description of HTS assay, compound library, screening conditions and post HTS analyses. (PDF) [file pcbi.1004153.s015.pdf]

Category	Parameter	Description
Assay	Type of assay	Cell-based
	Target	Cell cycle modulators
	Primary measurement	Cell cycle profile, detection of G2/M arrest
	Key reagents	Vybrant DyeCycle Green Stain (Invitrogen)<br>P-H3-488 Antibody (Cell Signaling)
	Assay protocol	See Extended Experimental Procedures
Library	Library size	90,000 compounds
	Library composition	Drug-like molecules
	Source	UCLA Molecular Screening Shared Resource
Screen	Format	384-well plates
	Concentration(s) tested	10μM, <1% DMSO
	Plate controls	Internal controls DMSO, Taxol
	Reagent/ compound dispensing system	Biomek FX (Beckman Coulter) and Multidrop 384 (Thermo LabSystems) liquid handlers
	Detection instrument and software	Acumen eX3 (TTP Labtech)
	Assay validation/QC	Z' score 0.4932 ± 0.0953
	Correction factors	
	Normalization	To internal controls DMSO and Taxol
Post-HTS analysis	Hit criteria	>70% G2/M arrest
	Hit rate	2.35%
	Additional assay(s)	Retesting compounds, counter screen for P-H3-488 antibody positives
	Confirmation of hit purity and structure	Compounds were repurchased from MolPort and compound structure and purity were verified analytically using LC/MS and NMR
