# Supplementary material for: Large-Scale Chemical Similarity Networks for Target Profiling of Compounds Identified in Cell-Based Chemical Screens
Source: PLoS Comput Biol. 2015 Mar 31;11(3):e1004153. doi: 10.1371/journal.pcbi.1004153 (PMC4380459; doi:10.1371/journal.pcbi.1004153)
Supplement: S4 Table — Five compounds (one from each of the five predicted target chemical similarity sub-networks) were selected for phenotypic analysis including compound 1 from the SCD sub-cluster (cluster 6), compound 2 that overlapped with both SCD and ABL1 sub-clusters (cluster 6) and compound 3 from the ABL1 sub-cluster (cluster 6). Additionally, compound 4 and compound 5, were retrieved from the PTPN cluster (cluster 3) and the TUBB cluster (cluster 4) respectively. Note that the reference ChEMBL compounds are in gray, the mitotic compounds are in red and the selected compounds are in yellow. (PDF) [file pcbi.1004153.s017.pdf]

compound ID	compound name	chemical structure	chemical similarity network	target	reference analogs
Compound **1**	N-{5-[(4-cyanophenyl)methyl]-1,3-thiazol-2-yl}-1,3-benzothiazole-6-carboxamide	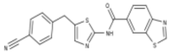	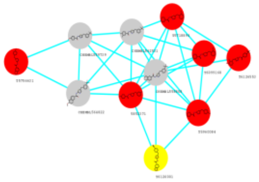	SCD	CHEMBL558808
Compound **2**	N-{5-[(2-chlorophenyl)methyl]-1,3-thiazol-2-yl}pyridine-4-carboxamide	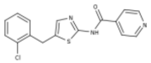	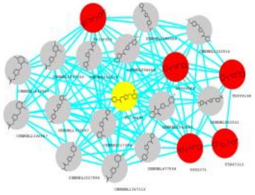	SCD/ABL1	SCD: CHEMBL558808, CHEMBL562531 ABL1: CHEMBL518430, CHEMBL517204, CHEMBL477554
Compound **3**	N-(5-benzyl-1,3-thiazol-2-yl)-3-chloro-1-benzothiophene-2-carboxamide	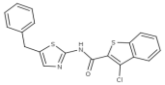	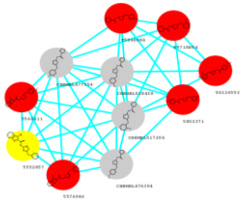	ABL1	CHEMBL477554, CHEMBL518430, CHEMBL517204, CHEMBL476304
Compound **4**	2-[(3-cyano-6-ethylquinolin-2-yl)sulfanyl]-N-(2,5-dimethylphenyl)acetamide	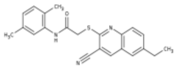	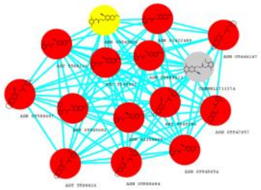	PTPN	CHEMBL1713374
Compound **5**	2-[(Z)-1-chloro-2-(3-methoxyphenyl)ethenyl]-6,7-dimethoxy-3,4-dihydroquinazolin-4-one	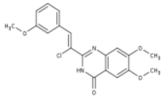	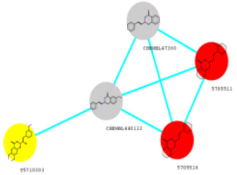	TUBB	CHEMBL440113
