# Supplementary material for: Large-Scale Chemical Similarity Networks for Target Profiling of Compounds Identified in Cell-Based Chemical Screens
Source: PLoS Comput Biol. 2015 Mar 31;11(3):e1004153. doi: 10.1371/journal.pcbi.1004153 (PMC4380459; doi:10.1371/journal.pcbi.1004153)
Supplement: S1 Text — (PDF) [file pcbi.1004153.s018.pdf]

Supporting Tutorials

Tutorial S1. Visualizing CSNAP results

1. Install the Cytoscape program (http://www.cytoscape.org/).
2. Start the program, select “file” then “open”, browse to the directory containing the Cytoscape files. Click on either “benchmark.cys” or “mitotic.cys” Cytoscape files.
3. Select desired networks, the compound attributes will appear in the lower panel when the network is selected (Figure A).

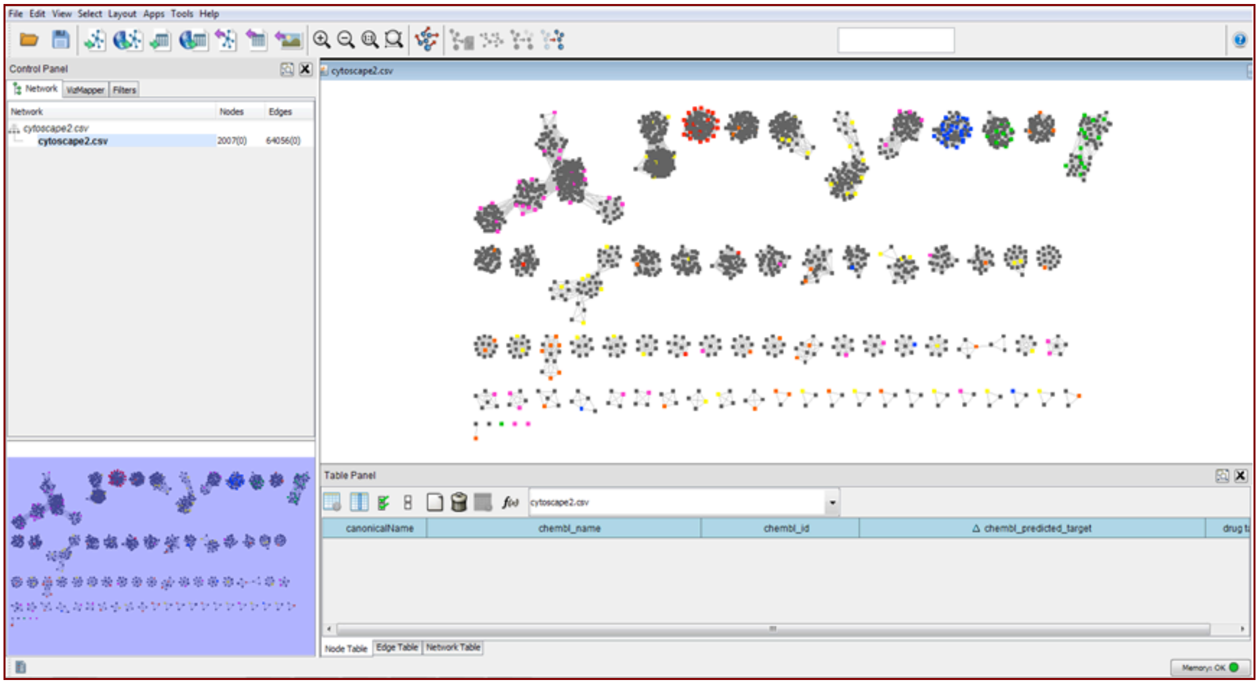

Figure A. CSNAP visualization on Windows operating systems.

Tutorial S2. CSNAP analysis of a PARP chemical similarity sub-network

1. In the second tutorial, we will study one chemical similarity sub-network consisting of PARP inhibitors from the benchmark sets. First, open the benchmark sets file “benchmark.cys” in the Cytoscape program as instructed from the previous step.
2. To facilitate target analysis, we will focus on the target prediction based on the ChEMBL database. To do this, click on the “show column” icon below the table panel then check the following four node attributes: “name”, “chembl_name”, “chembl_id” and “chembl_predicted_target”. Meanwhile, uncheck any unwanted columns. The column position can be freely arranged by mouse dragging action.
3. Select the blue chemical network cluster as shown (Figure B). The chemical similarity network can be zoomed-in using mouse scrolling action. The global location of the network can be determined from the lower left panel.

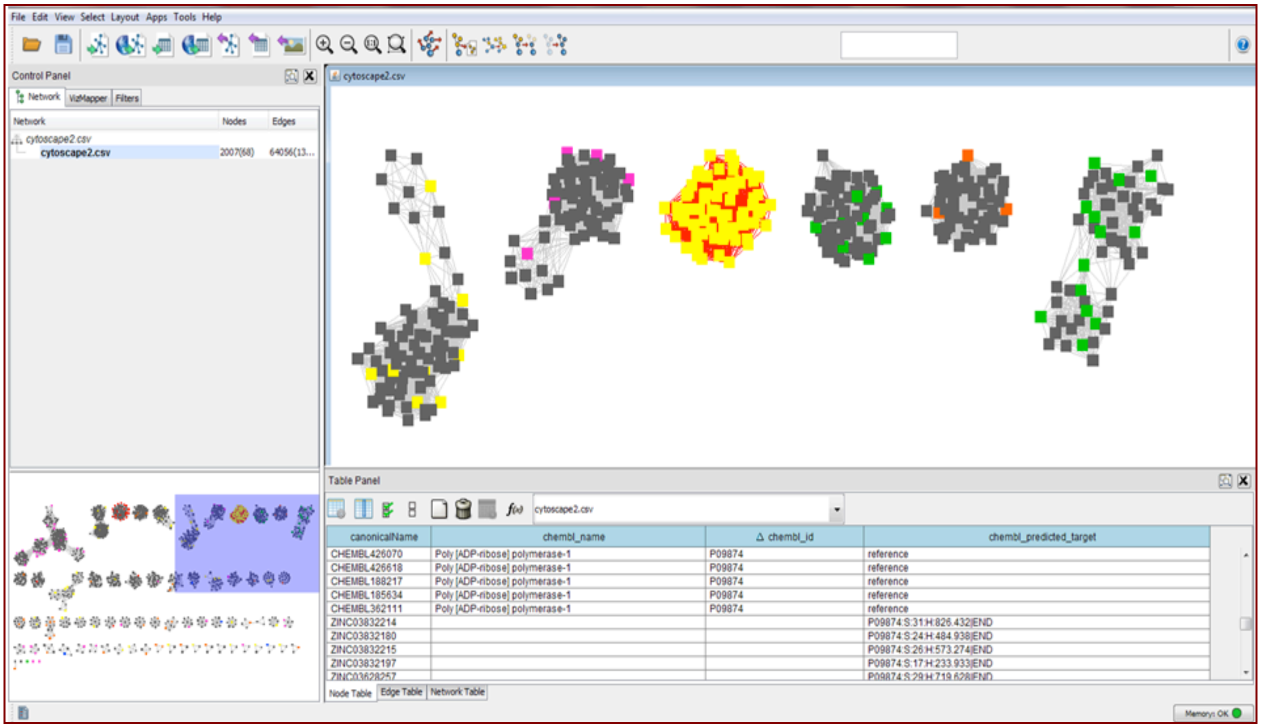

Figure B. CSNAP analysis of a PARP chemical similarity sub-network.

4. Figure C shows a partial view of the table consisting of the selected columns for the PARP chemical similarity sub-network. For reference ChEMBL compounds, the target name (chembl_name) and their associated target Uniprot ID (chembl_id) are labeled. Since there is no predicted target for reference compounds, their "chembl_predicted_target" columns are labeled as "reference".

canonicalName	chembl_name	Δ chembl_id	chembl_predicted_target
CHEMBL362568	Poly [ADP-ribose] polymerase-1	P09874	reference
CHEMBL361447	Poly [ADP-ribose] polymerase-1	P09874	reference
CHEMBL361489	Poly [ADP-ribose] polymerase-1	P09874	reference
CHEMBL188308	Poly [ADP-ribose] polymerase-1	P09874	reference
CHEMBL426977	Poly [ADP-ribose] polymerase-1	P09874	reference
CHEMBL361675	Poly [ADP-ribose] polymerase-1	P09874	reference
CHEMBL187566	Poly [ADP-ribose] polymerase-1	P09874	reference
CHEMBL189173	Poly [ADP-ribose] polymerase-1	P09874	reference
CHEMBL189670	Poly [ADP-ribose] polymerase-1	P09874	reference
CHEMBL188989	Poly [ADP-ribose] polymerase-1	P09874	reference
CHEMBL188728	Poly [ADP-ribose] polymerase-1	P09874	reference
CHEMBL187344	Poly [ADP-ribose] polymerase-1	P09874	reference
CHEMBL186461	Poly [ADP-ribose] polymerase-1	P09874	reference
CHEMBL426070	Poly [ADP-ribose] polymerase-1	P09874	reference
CHEMBL426618	Poly [ADP-ribose] polymerase-1	P09874	reference
CHEMBL188217	Poly [ADP-ribose] polymerase-1	P09874	reference
CHEMBL185634	Poly [ADP-ribose] polymerase-1	P09874	reference
CHEMBL362111	Poly [ADP-ribose] polymerase-1	P09874	reference
ZINC03832214			P09874:S:31:H:826.432|END
ZINC03832180			P09874:S:24:H:484.938|END
ZINC03832215			P09874:S:26:H:573.274|END
ZINC03832197			P09874:S:17:H:233.933|END
ZINC03628257			P09874:S:29:H:719.628|END
ZINC03832187			P09874:S:16:H:205.462|END
ZINC03832192			P09874:S:17:H:233.933|END
ZINC03832216			P09874:S:27:H:620.212|END
ZINC03832178			P09874:S:31:H:826.432|END
ZINC03832211			P09874:S:30:H:772.107|END
ZINC03832201			P09874:S:29:H:719.628|END
ZINC03832185			P09874:S:19:H:296.415|END

Figure C. Partial table showing ChEMBL target annotation and scoring.

5. For reference ZINC compounds, the CHEMBL predicted targets are shown in the "chembl_predicted_target" columns. The output format represents a rank list of two target prediction scores: Schwikowski scores (S-score) and Hishigaki scores (H-score) in decreasing order :

[uniprot ID1, S-score1, H-score1 |…|uniprot IDN, S-scoreN, H-scoreN | END]

6. For all selected query ZINC compounds within the chemical similarity sub-network, the consensus predicted Uniprot ID is P09874, corresponding to the protein target Poly [ADP-ribose] polymerase-1 with S-scores ranging from 12 to 33. To check the pre-labeled target of the query compounds, click "show column" then check "drug target". This should reveal that the selected queries are known PARP binders. Thus, the CSNAP approach has correctly predicted the drug target of these PARP inhibitors.

7. To gain additional understanding of the S-score, select compound node “ZINC03832182” with S-score of 12, indicating that there are 12 ChEMBL reference nodes in its first neighbor. To see this, click “First Neighbors of Selected Nodes” icon on the top panel then click “New Network From Selections”.
8. As shown in Figure D, there are 12 CHEMBL nodes connecting to the selected compound “ZINC03832182”.

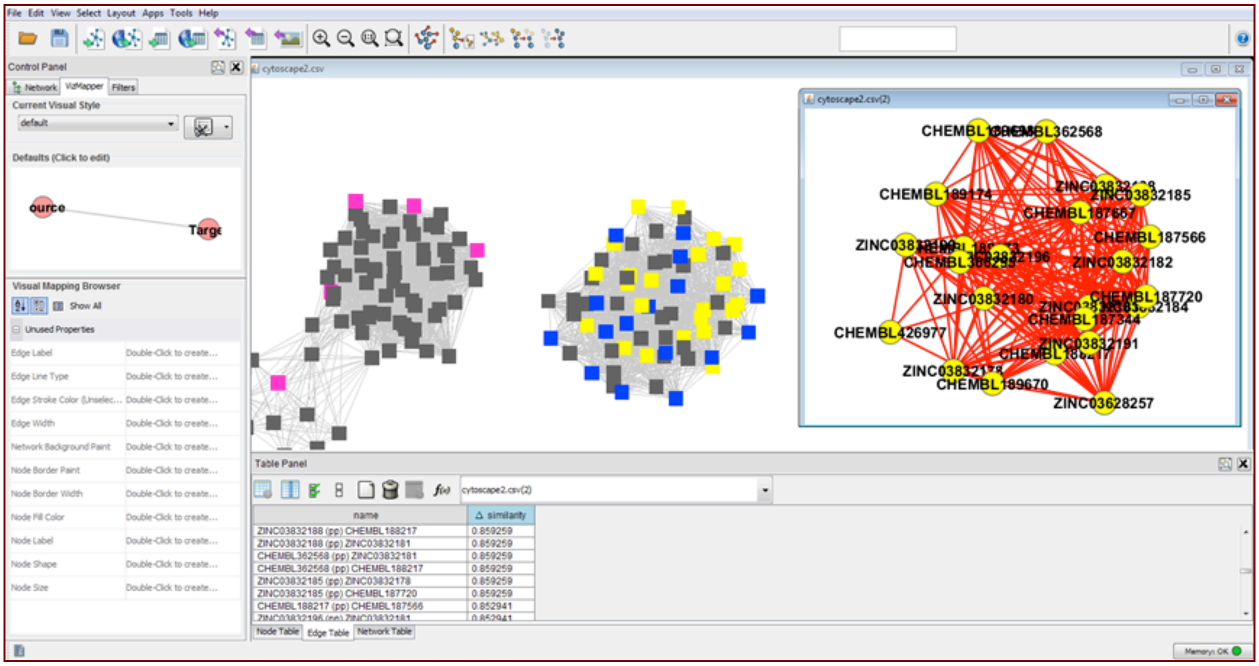

Figure D. Neighborhood analysis of PARP compounds.

9. To further identify the similarity between clustered ligands, switch to the edge view by clicking the “Edge Table” tab on the bottom panel. Next, click “Show Column” then check both “Name” and “Similarity” edge attributes. Finally, click the desired edge on the chemical similarity sub-network. Figure D shows that PARP compounds were clustered with similarity values ranging from 0.7 to 1.
10. The analysis described will be generally applicable to any chemical similarity network analysis for drug target prediction using CSNAP. This concludes the CSNAP tutorials.

Supporting Materials and Methods

CSNAP Performance Comparison

We compared CSNAP target prediction accuracy with SEA (similarity ensemble approach), a commonly used ligand-based approach that has been validated experimentally for phenotypic-based target discovery [1]. The validation set of 206 known drug classes and bioactivity database (ChEMBL version 12) were used to search the SEA server using default parameters and the target prediction results generated by the server were compared to those generated by CSNAP analysis (Extended Experimental Procedures and Table S1). Overall, CSNAP provided a substantial improvement in target prediction accuracy over the SEA approach in correctly identifying the labeled targets of six drug classes from the top hits (80% versus 63%), the top five hits (92% versus 73%) and the top ten hits (94% versus 75%) respectively (Figure 3A). While the accuracy for target specific drugs (HMGA, HSP90 and PARP) were comparable between the two approaches, CSNAP had a greater success rate at identifying the main target of promiscuous ligands such as CDK2 (92% versus 30%) and ACE (96% versus 65%) inhibitors, which usually results in low prediction accuracies by traditional ligand-based methods (Figures 3B and 3C).

LTIF Off-target Analysis of Mitotic Set

By analyzing LTIF off-target binding patterns of each predicted drug class, the heat maps of predicted SCD and ABL1 inhibitors showed several overlapping peaks, indicating that several compounds were targeting both SCD and ABL1 (Figure S4). As expected, CSNAP analysis indicated that both the SCD and ABL1 compound sets mapped onto the same chemical similarity

sub-network from which 4 potential dual inhibitors targeting both SCD and ABL1 were identified (cluster 6) (Figure 4C). In contrast, PTPN (cluster 3) and TUBB (clusters 4 and 5) were represented by distinct chemical similarity clusters, suggesting high target specificity for these predicted compounds.

Derivation of Mitotic Set Compounds

The 212 antimitotic compounds analyzed by CSNAP were identified through a cell-cycle chemical screening approach as described in (Senese et al., submitted). Briefly, HeLa cells plated in 384-well plates (1500 cells/well) and treated with 10μM drugs for 20 hours followed by fixation and staining with 5μM Vybrant DyeCycle Green (Invitrogen) for 1 hour at room temperature. Compound-induced cell cycle profiles were generated by scanning the plates with an Acumen eX3 (TTP Labtech) fluorescence cytometer. Compounds with a >67% G2/M arrest were selected for further analysis. To determine if these compounds were arresting cells in G2-phase or M-phase, cells were fixed with 4% paraformaldehyde, permeabilized with 0.2% Triton X-100/PBS and stained with Alexa-488-phospho-histone-H3 (Ser10, Cell Signaling) and 1μg/ml Hoechst 33342 for 1 hour following 20 hours compound addition. Plates were then imaged with an ImageXpress Micro (Molecular Devices) high-content fluorescence microscope and the percent mitotic index was quantified using the CDD (Collaborative Drug Discovery) software.

MitoCheck Database Search

To validate that the predicted protein targets had mitotic specific functions and to determine their mitotic phenotypes, we queried the MitoCheck database, which maintains data on the mitotic

phenotypes observed upon siRNA knockdown of gene expression for almost all human genes[2]. Among the 20 predicted targets, depletion of 14 induced mitotic defects and no data was available for 2, giving a hit rate of 70% (Figure S6). Because compounds that modulate one target family may also modulate related families by structural homology, we subsequently queried all the subtypes within each target category (Figure S7). This analysis showed that all four target categories (SCD, ABL1, PTPN and TUBB) displayed diverse mitotic phenotypes by siRNA knockdown. The identified phenotypes included mitotic delay, post-mitotic defects (binuclear and polylobed nucleus) and apoptosis (cell death), suggesting that these targets were essential for cell division and their knockdowns led to defects in spindle assembly, chromosome segregation problems and hindrance of cytokinesis (Figures S6 and S7) [2].

Deconvolution of Mitotic Compounds Targeting Microtubules

To test CSNAP's prediction that 51 of the 212 mitotic compounds were targeting microtubules, we re-acquired all 212 compounds to verify their purity/structural identity and tested their ability to perturb microtubule polymerization (stabilize or destabilize microtubules) in an *in-vitro* microtubule polymerization assay at 50μM concentration (Figure 5A). The end-point absorbance (dOD) was used to quantify the degree of microtubule polymerization and was converted to percentage fold change (F) relative to DMSO drug vehicle (0%), as previously described (Figure 5A) [3]. As additional controls, several known tubulin stabilizers and destabilizers were tested including paclitaxel (5 μM) (F= +103.3%), vinblastine (5 μM) (F= -74.65%), colchicine (50 μM) (F= -106.45%) and fenbendazole (50 μM) (F= -27.11%). From this, we set the criteria of F > 40% for strong stabilizers/destabilizers, F=20-40% for weak stabilizers/destabilizers and F< 20% for no effect on tubulin polymerization (Figure 5A and Table S3).

Network SAR Analysis of Microtubule Destabilizers

A chemical similarity sub-network consisting of 7 novel anti-tubulin ligands based on a phenyl-sulfanyl-thiazol-acetamide scaffold were analyzed. All 7 compounds showed a microtubule destabilizing effect. By traversing the network through a path of minimum structural change starting with compound **6** (F=33.1%), increasing the hydrophobicity in the northern part of the molecule by substituting the N-methyl group (within the triazole scaffold) with either a propyl or phenyl group in compounds **7** (F=56.9%) and **8** (F=50.9%) enhanced the microtubule destabilizing effect (Figure 5B). In contrast, a chemical "knock-down" that replaced the larger amido-phenyl rings in the eastern region of compounds **8** and **9** (F=57.2%) with smaller amido-thiazole rings in compounds **10** (F=40.1%), **11** (F=51%) and **12** (F<20%) led to an overall decrease in the destabilizing effect.

Determination of Compound Potency in Cell Culture

We asked if the observed *in-vitro* SAR was reproducible in living cells. To do this, we evaluated each compound's potency (EC_{50}) in HeLa cells with regards to their ability to arrest cells in G2/M phase and to induce cell death. HeLa cells were treated with a 20-point titration (0-100 μM) of each drug and their $EC_{50,\ G2/M}$ and $EC_{50,\ cell\ death}$ were quantified by analyzing their cell cycle profiles and measuring total ATP levels at 20 and 72 hours post-treatment respectively (Figure S10 and Extended Experimental Procedures). Consistently, a similar SAR trend was observed in cell culture. The addition of an N-phenyl group on the triazole base contributed to an overall increase in compound potency ranging from low micro-molar for compounds **6**

($EC_{50:G2/M}$ = 1.53 μM; $EC_{50: cell death}$ = 1 μM) and **7** ($EC_{50:G2/M}$ = 3.34 μM; $EC_{50: cell death}$ = 3.38 μM) to nano-molar ranges for compounds **8** ($EC_{50:G2/M}$ = 33 nM; $EC_{50: cell death}$ = 60 nM) and **9** ($EC_{50:G2/M}$ = 550 nM; $EC_{50: cell death}$ = 550 nM). Likewise, potency increased for compounds substituted with an amido-phenyl ring including compounds **8** and **9** in contrast to those with an amido-triazole ring including compounds **10** ($EC_{50:G2/M}$ = 1.59 μM; $EC_{50: cell death}$ = 1.41 μM), **11** ($EC_{50:G2/M}$ = 784 nM; $EC_{50: cell death}$ = 545 nM) and **12** ($EC_{50:G2/M}$ = 973 nM; $EC_{50: cell death}$ = 883 nM). Thus the most potent compound was **8**.

In-vitro Mass Spectrometry Competition Binding Assay

To verify the predicted compound-binding model, we competed the colchicine-tubulin interaction with compound **8**, the most potent compound in the series, using a competitive mass spectrometry binding assay by analyzing the filtrate of unbound ligands with LC-MS/MS. The results indicated that compound **8** competed strongly for the colchicine site where only (5.7 ± 7.8) % of colchicine remained bound compared to the negative control vincristine where (88.4% ± 2.2) % of colchicine remained bound (Figure 5F).

CSNAP Protocol

(A) Target identification. Chemical similarity comparison was performed between query compounds and ChEMBL reference compounds using FP2 fingerprints in the Obabel program version 2.3.1[4]. From this, any ChEMBL reference compounds with Tc-cutoff > 0.85 or Z-score > 2.5 were selected as baits. The Z-score is defined by the standard deviation from the mean of the top 100 compounds Tc similarity score distribution. To create network edges connecting

compound nodes, an adjacency similarity matrix was computed between every compound pair (both query and bait nodes) by removing any similarity edges with Tc threshold< 0.7. This created a weighted graph (chemical similarity network) with nodes representing compounds and edges representing chemical similarity.

(B) Bioactivity database search. The bioactivities data from the ChEMBL database including target name, target confidence score, organism, assay type, target ChEMBL ID, and activity comment, and from the PubChem database including stdInChIKey, assay ID, protein UniProt ID, protein name, gene ID and gene symbol were retrieved dynamically using the RESTful (Representational State Transfer) web service and parsed in JSON (Javascript Object Notation) format. For the ChEMBL bioactivities, the target annotations were further filtered using ChEMBL confidence scores, which assess the confidence that the target is assigned correctly to a particular assay. In this scoring scheme, we retained target annotations with a confidence score >= 4, indicating that a specific target has been identified for the assay. Additionally, three types of assays are defined in the ChEMBL database: Biochemical assay (B), Functional assay (F) and ADMET assay (A). Subsequently, the processed target annotations were further filtered by the assay type and retained type (B), indicating a direct protein-ligand interaction. For the PubChem bioactivities, target annotations were assessed based on activity comments and those labeled as "active" were retained. All the data processing has been automated using shell and perl programming language.

(C) Target assignment and scoring. The generated CSNAP network topology was used to guide and quantify the target assignment using two neighbor counting functions including

Schwikowski score (S-score) and Hishigaki score (H-score) as previously described for protein functional prediction in protein-protein interaction (PPI) network [5,6]. Specifically, for each query compound in the network, the target annotation frequencies in the reference nodes of its first-order neighbor were counted and the most probable targets for each query were ranked and identified. The S-score provided a weighted measure for each predicted targets for a given query compound in the CSN. To further provide a statistical measure for the S-score, an alternative scoring scheme, H-score was used to evaluate a significant score for each S-score assignment [7]. The Hishigaki score is a chi-square like test according to the formula:

$$\chi_i^2 = \frac{(N_i - E_i)^2}{E_i}$$

where i represents an assigned target, Ei represents the expected number of assigned targets i in the first-order neighbor of all query compounds distributed within the similarity network, and Ni represents the observed frequency of assigned targets i in the first-neighborhood of the specific query under consideration[7].

CSNAP Analysis

(A) Network visualization. The CSNAP outputs were visualized using the Cytoscape program version 2.8.2 (Supplemental Tutorials)[8]. The output data was imported in csv file format, where compounds were represented by nodes and similarity was represented by edges. The node attributes included the Simplified Molecular Input Line Entry System (SMILES) notation, InChIKey, predicted target ID, predicted target name and the predicted target score. Duplicate edges and self-loops were removed using the “network modification” plugin. Additionally, the network configuration was reordered using the “force-direct layout” based on ligand similarity

value. For the prepared data set, see the Supplemental File.

(B) Hit enrichment by S-core filter. We sought a way to optimize CSNAP for hit enrichment by reducing the number of retrieved target IDs while maintaining its accuracy, which would be useful for target virtual screening and validation. To do this, we filtered the 740 UniProt target IDs using two S-score cutoffs (5 and 10) and determined the final target ID numbers and prediction accuracy. By comparing percentage target number reduction and overall target prediction accuracy, we identified an optimal S-score cutoff of 4, by the interaction of two curves. Further application of this cut-off had reduced the total number of candidate IDs to 89 while maintaining an overall accuracy of 80%.

(C) Generation of ligand target interaction fingerprint (LTIF). To analyze off target effects of the query compound, LTIF were created as previously described [9]. The predicted S-score for predicted targets of each compound from the CSNAP analysis were mapped against all the predicted targets, resulting in an M x N matrix where M is the number of compounds and N is the number of selected targets. The LTIF matrix were generated and visualized as a heat map using the "heatmap" function in the R statistical package.

Target Prediction Accuracy Comparison of CSNAP and SEA Approaches

The 206 benchmark compounds in SMILES notation were used to query the SEA server (http://sea.bkslab.org/) with default parameters (CHEMBL version 12 binding, Scitegic ECFP4 fingerprint) and the predicted targets were ranked and identified by the E-values. If the labeled

targets of each query compound were not identified from the top hit, the rank of the labeled target and its associated E-value were identified from the ranking list. To enable valid comparison, the same validation compounds were re-analyzed by CSNAP using CHEMBL version 12 and the top predicted targets were ranked by the S-score as described previously. Similarly, if the labeled targets were not identified from the top hits, the rank of the labeled target and its associated S-score were identified from the ranking list. Finally, the prediction accuracies (% of correctly predicted ligands) of the two approaches were compared. For complete comparison results, please see Figure 3 and Table S1.

Determination of Compound Potency in Cell Culture

To determine the potency of compounds **6-12** in cell culture**,** HeLa cell were treated with increasing concentrations (20-point titration 0-100 μM) of indicated compounds and evaluated by two cell-based assays. For the cell cycle arrest assay, HeLa cells were treated with indicated compounds for 20 hours and the extent of cell cycle arrest was quantified using the DNA intercalating agent Vybrant DyCycle Green and the percentage of cells in G2/M was quantified with an Acumen eX3 (TTP Labtech) high-content screening cytometer using its 488 nm laser. For cell viability assays, cells were treated with compounds for 72 hours and the extent of cell death was quantified using the CellTiter-Glo assay (Promega) with a Tecan M1000 multi-well plate reader at OD 540 nm. The EC_{50s} were then calculated using the CD software.

Supporting References

1. Laggner C, Kokel D, Setola V, Tolia A, Lin H, et al. (2012) Chemical informatics and target identification in a zebrafish phenotypic screen. Nat Chem Biol 8: 144-146.
2. Neumann B, Walter T, Heriche JK, Bulkescher J, Erfle H, et al. (2010) Phenotypic profiling of the human genome by time-lapse microscopy reveals cell division genes. Nature 464: 721-727.
3. Hu W, Dong H, Li YZ, Hu XT, Han GJ, et al. (2004) A high-throughput model for screening anti-tumor agents capable of promoting polymerization of tubulin in vitro. Acta Pharmacol Sin 25: 775-782.
4. O'Boyle NM, Banck M, James CA, Morley C, Vandermeersch T, et al. (2011) Open Babel: An open chemical toolbox. J Cheminform 3: 33.
5. Schwikowski B, Uetz P, Fields S (2000) A network of protein-protein interactions in yeast. Nat Biotechnol 18: 1257-1261.
6. Sharan R, Ulitsky I, Shamir R (2007) Network-based prediction of protein function. Mol Syst Biol 3: 88.
7. Hishigaki H, Nakai K, Ono T, Tanigami A, Takagi T (2001) Assessment of prediction accuracy of protein function from protein--protein interaction data. Yeast 18: 523-531.
8. Shannon P, Markiel A, Ozier O, Baliga NS, Wang JT, et al. (2003) Cytoscape: a software environment for integrated models of biomolecular interaction networks. Genome Res 13: 2498-2504.
9. Gregori-Puigjane E, Mestres J (2008) A ligand-based approach to mining the chemogenomic space of drugs. Comb Chem High Throughput Screen 11: 669-676.
